# Cancer-associated fibroblast-specific lncRNA LINC01614 enhances glutamine uptake in lung adenocarcinoma

**DOI:** 10.1186/s13045-022-01359-4

**Published:** 2022-10-08

**Authors:** Tongyan Liu, Chencheng Han, Panqi Fang, Zhifei Ma, Xiaoxiao Wang, Hao Chen, Siwei Wang, Fanchen Meng, Cheng Wang, Erbao Zhang, Guozhang Dong, Hongyu Zhu, Wenda Yin, Jie Wang, Xianglin Zuo, Mantang Qiu, Jinke Wang, Xu Qian, Hongbing Shen, Lin Xu, Zhibin Hu, Rong Yin

**Affiliations:** 1 Department of Thoracic Surgery, Jiangsu Key Laboratory of Molecular and Translational Cancer Research, Jiangsu Cancer Hospital and Nanjing Medical University Affiliated Cancer Hospital and Jiangsu Institute of Cancer Research, Nanjing, 21009 People’s Republic of China; 2 Department of Science and Technology, Jiangsu Cancer Hospital and Nanjing Medical University Affiliated Cancer Hospital and Jiangsu Institute of Cancer Research, Nanjing, 21009 People’s Republic of China; 3 Department of GCP Research Center, Jiangsu Province Hospital of Chinese Medicine, The Affiliated Hospital of Nanjing University of Chinese Medicine, Nanjing, 210029 People’s Republic of China; 4 Collaborative Innovation Center for Cancer Personalized Medicine, Nanjing Medical University, Nanjing, 211116 People’s Republic of China; 5 Department of Epidemiology and Biostatistics, International Joint Research Center On Environment and Human Health, Center for Global Health, School of Public Health, Nanjing Medical University, Nanjing, 211116 People’s Republic of China; 6 Department of Bioinformatics, Nanjing Medical University, Nanjing, 211166 People’s Republic of China; 7 Biobank of Lung Cancer, Jiangsu Biobank of Clinical Resources, Nanjing, 21009 People’s Republic of China; 8 Department of Thoracic Surgery, Peking University People’s Hospital, Beijing, 100044 People’s Republic of China; 9 State Key Laboratory of Bioelectronics, Southeast University, Nanjing, 210018 People’s Republic of China

**Keywords:** Tumor microenvironment, Cancer-associated fibroblasts, Long noncoding RNA, Metabolic reprograming, Glutamine

## Abstract

**Background:**

Besides featured glucose consumption, recent studies reveal that cancer cells might prefer “addicting” specific energy substrates from the tumor microenvironment (TME); however, the underlying mechanisms remain unclear.

**Methods:**

Fibroblast-specific long noncoding RNAs were screened using RNA-seq data of our NJLCC cohort, TCGA, and CCLE datasets. The expression and package of LINC01614 into exosomes were identified using flow cytometric sorting, fluorescence in situ hybridization (FISH), and quantitative reverse transcription polymerase chain reaction (RT-PCR). The transfer and functional role of LINC01614 in lung adenocarcinoma (LUAD) and CAFs were investigated using 4-thiouracil-labeled RNA transfer and gain- and loss-of-function approaches. RNA pull-down, RNA immunoprecipitation, dual-luciferase assay, gene expression microarray, and bioinformatics analysis were performed to investigate the underlying mechanisms involved.

**Results:**

We demonstrate that cancer-associated fibroblasts (CAFs) in LUAD primarily enhance the glutamine metabolism of cancer cells. A CAF-specific long noncoding RNA, LINC01614, packaged by CAF-derived exosomes, mediates the enhancement of glutamine uptake in LUAD cells. Mechanistically, LINC01614 directly interacts with ANXA2 and p65 to facilitate the activation of NF-κB, which leads to the upregulation of the glutamine transporters SLC38A2 and SLC7A5 and eventually enhances the glutamine influx of cancer cells. Reciprocally, tumor-derived proinflammatory cytokines upregulate LINC01614 in CAFs, constituting a feedforward loop between CAFs and cancer cells. Blocking exosome-transmitted LINC01614 inhibits glutamine addiction and LUAD growth in vivo. Clinically, LINC01614 expression in CAFs is associated with the glutamine influx and poor prognosis of patients with LUAD.

**Conclusion:**

Our study highlights the therapeutic potential of targeting a CAF-specific lncRNA to inhibit glutamine utilization and cancer progression in LUAD.

**Supplementary Information:**

The online version contains supplementary material available at 10.1186/s13045-022-01359-4.

## Introduction

Metabolic reprogramming is a hallmark of cancer, which endows cancer cells with growth and proliferative potential under nutrient-deprivation tumor microenvironment (TME) conditions [1]. The founding observation in cancer metabolism was the use of aerobic glycolysis, wherein glucose is mainly processed into lactate [2]. Beyond glycolysis, cancer cells also use glutaminolysis and fatty acid oxidation to support their biosynthetic demands [3]. Recently, studies have revealed that glucose is preferentially consumed by immune cells (than cancer cells), whereas cancer cells exhibited the highest glutamine uptake, highlighting the probability that cancer cells conditionally prefer specific “addicting” nutrients in the TME [4, 5]. However, little is known about intrinsic mechanisms that facilitate the addiction phenomenon of cancer cells.

Recent studies have demonstrated that one of the abundant stromal components in the TME, i.e., cancer-associated fibroblasts (CAFs), is important in regulating cancer cell metabolism and acts as potential cancer therapeutic targets [6]. Previous findings have demonstrated that the metabolic intermediates and ATP produced by CAFs support the growth of adjacent cancer cells [7–9]. High FAK-expressing CAFs enhance glycolysis of breast and pancreatic cancer cell by increasing chemokine production [10]. However, whether and how CAFs facilitate a preference for specific “addicting” nutrients remain unclear in lung cancer.

In the present study, transcriptomic and metabolomic analyses indicated that CAFs mainly regulate the amino acids of lung adenocarcinoma (LUAD) cells in an exosome-dependent manner. We also found that CAF-derived exosomes preferentially enhance glutamine uptake in LUAD cells. Mechanistically, exosome-packaged long noncoding RNAs (lncRNAs) are shown to play a critical role in mediating crosstalk between cancer cells and TME [11]. More importantly, because the expression of lncRNAs is remarkably tissue and cell type-specific [12–14], the discovery of lineage and TME-specific lncRNAs in exosomes mediating crosstalk between CAFs and LUAD cells may provide further insights regarding their roles in cancer progression. Because of the limitation of scRNA-seq with respect to identifying noncoding transcripts, we screened and validated non-tumor expressed lncRNAs in TME populations based on the Nanjing Lung Cancer Cohort (NJLCC) [15] and The Cancer Genome Atlas (TCGA)-LUAD dataset, and eventually examined their presence in CAF-derived exosomes. We identified LINC01614 as a CAF-specific lncRNA and demonstrated that CAF-exosome-packaged LINC01614 could regulate the glutamine metabolism of cancer cells. We further dissected the mechanisms underlying these actions.

## Methods and materials

### Primary CAF isolation from LUAD

Human primary fibroblasts were isolated from fresh LUAD tissues (CAFs) and adjacent non-tumor tissues (NFs). Samples were collected after surgical resection of the tissue storage buffer (Miltenyi) and washed in phosphate-buffered saline (PBS) containing 1% antibiotic–antimycotic (Gibco, Life Technologies). The tissues were minced into small (1–2 mm) pieces and digested with a Human Tumor Dissociation Kit using gentleMACS Octo Dissociator following the manufacturer’s instructions (Miltenyi). The digested samples were sequentially filtered through 70 μm cell strainers. The cells were then collected by centrifugation at 250 g for 5 min and grown in Dulbecco's Modified Eagle Medium (DMEM) (Gibco, Carlsbad, CA, USA) supplemented with 10% fetal bovine serum (FBS) and 1% antibiotic–antimycotic at 37 °C. The medium was changed every 3 d. Fibroblasts were obtained using a differential time adherent method, as previously reported [16]. The purity of fibroblasts was evaluated with flow cytometry analysis and immunofluorescent staining. Primary CAFs were negative for EpCAM, CD45, and CD31 and positive for FAP and α-SMA. Cells were used up to 10 passages. The clinical information for patients whose tumors were used for CAF and NF isolation is listed in Additional file 1: Table S1. All samples were obtained from the donors with informed consent, and all related procedures were conducted with the approval of the Ethics Committee of Jiangsu Cancer Hospital.

To knock down or overexpress specific genes, primary fibroblasts were transduced with the lentiviral vectors (multiplicity of infection [MOI] of 100) at 37 °C with 5 μg mL^−1^ polybrene (Sigma). The targeting sequences of each shRNA are provided in Additional file 1: Table S2.

### Cell culture, cell proliferation, migration, and invasion assays

All cell lines (A549, SPC-A1, H1975, H827, H2228, PC9, MRC5, and human bronchial epithelial (HBE) cells) were obtained from Shanghai Institutes for Biological Science (Shanghai, China). In a 6-well Transwell system, A549 or H1975 cells (1 × 10^6^) were plated on the lower chamber, whereas CAFs (1 × 10^6^) were added to the upper chamber; the pore size was 0.4 μm. All cells were grown in DMEM supplemented with 10% fetal bovine serum (FBS), 50 U mL^−1^ penicillin (Gibco), and 50 μg mL^−1^ streptomycin (Gibco). All cells were authenticated and tested routinely for their authenticity and free from mycoplasma contamination.

Cell proliferation was detected using a Real-time xCELLigence analysis system (RTCA) according to the manufacturer’s protocol (ACEA Biosciences). Migration assay of LUAD cells was performed in an 8 μm 24-well Boyden chamber (Millipore). Corning BioCoat Matrigel Invasion Chambers with 8 μm PET membranes (Corning) were used for invasion assays. LUAD cells (2 × 10^4^) suspended in 100 μL FBS-free DMEM were added to the upper chamber. 1 × 10^6^ CAFs concentrated in 750 μL of DMEM containing 10% FBS were added to the bottom of a 24-well plate. LUAD cells were allowed to migrate or invade for 24 h and 48 h, respectively. Quantification was performed by counting the mean number of cells in three microscopy fields per chamber. For a specific signaling pathway study, cells were pretreated with the vehicle (DMSO) and 6 μM JSH-23 or 10 μM BMS-345541 for 1 h at 37 °C prior to the experiments.

### Tissue samples and microarrays

All primary LUAD tissues and adjacent normal tissues were obtained from 154 patients who underwent surgery at the Affiliated Cancer Hospital of Nanjing Medical University (Jiangsu Cancer Hospital, Nanjing, China). Tissue samples were used to quantify LINC01614, α-SMA, SLC38A2, and SLC7A5 and Kaplan–Meier survival analysis. A total of 78 pairs of LUAD and adjacent normal tissues from the JSCH cohort were used to construct the TMA, as described previously [17]. All samples were reviewed by experienced pathologists and performed in accordance with the International Ethical Guidelines for Biomedical Research Involving Human Subjects. All samples were collected from patients with informed consent, and all related procedures were conducted with the approval of the Ethics Committee of Jiangsu Cancer Hospital (approval Number: No. 2018(83), 2018(107)).

### TCA metabolites and ATP content assays

LUAD cells were cultured to ~ 40% confluency and changed with the conditional medium. After 24 h, the cells were collected, and measurement of TCA metabolites (glutamate, succinate, and fumarate) was performed using kits from Biovision (catalogue nos. ab). An Enliten ATP assay system (Promega) was used to measure the ATP content of LUAD cells according to the manufacturer’s instructions.

### Glutamine consumption and uptake assays

The consumption rate of extracellular glutamine was measured with high-performance liquid chromatography (HPLC) after pre-column derivation with o-phthaldialdehyde (OPA) with an analytical column (C18; 4.6 mm × 15 cm, 3 μm, Sigma) and a guard column (C18; 4.6 mm × 5 cm, 15 μm, Waters). The glutamine in the medium was separated and quantified in the chromatogram. For the glutamine uptake assay, 1 × 10^5^ cells were seeded in 24-well plates 24 h before the experiment. Cells were incubated in medium supplemented with radiolabeled ^3^H-glutamine (0.1 μM) and ice-cold glutamine (50 μM) or background medium containing ^3^H-glutamine (0.1 μM) and glutamine (10 mM) at 37 °C, with 5% CO_2_ for 10 min. The cell lysates were then analyzed by liquid scintillation with a scintillation counter, and the background medium counts were subtracted. CPM values were normalized using μg of protein.

### Glucose consumption and lactate production assays

LUAD cells were cultured until 50% of confluency and then changed to a fresh culture medium. After 24 h, the supernatants were harvested and used for glucose consumption and lactate production tests. Glucose and lactate detection kits were purchased from Biovision (catalog nos. ab136955 and ab65331, respectively). All experiments were conducted according to the manufacturer’s instructions. The values were normalized using μg of protein.

### Seahorse assay

An XF24 Extracellular Flux Analyzer (Seahorse Bioscience) was applied to evaluate the OCR and ECAR. Cells were seeded in 96-well plates at a density of 8 × 10^3^ cells per well in a growth medium overnight. For OCR analysis, cells were washed and incubated with a mito stress-test base medium containing 10 mM glucose, 2 mM L-glutamine, 1 mM pyruvate. After every three measurements at 8 min intervals, 1 μM oligomycin, 3 μM FCCP, or 0.5 μM rotenone was added to the wells at the indicated time points. For ECAR analysis, cells were added with base medium with 2 mM L-glutamine and monitored every 3 min following successive administration of 10 mM glucose, 1 μM oligomycin, and 50 mM 2-deoxyglucose.

### Nontargeted metabolomics analysis

Metabolite extraction from CM was conducted according to previously reported methods [18]. Briefly, precooled methanol was added to the samples to extract the metabolites. After rotating for 1 min and incubating at −20 ℃ for 2 h, the samples were centrifuged for 20 min at 4000 rpm, the supernatant was collected, and it was transferred to autosampler vials for LC–MS analysis.

### Exosome experiments

Exosomes from CAFs culture medium were isolated through standard centrifugation steps or an Exosome Precipitation Solution. Briefly, the culture medium was centrifuged at 3000 rpm for 10 min at 4 °C, followed by centrifugation at 10,000×*g* for 30 min at 4 °C to remove cellular debris. The supernatant was then filtered using a 0.22 μm filter. Exosomes were isolated using an Exosome Precipitation Solution (ExoQuick-TC, System Biosciences) or centrifuged at 100,000×*g* for 90 min. Exosomes were examined by electron microscopy and quantified using a NanoSight NS300 instrument (Malvern Instruments) equipped with NTA 3.0 analytical software (Malvern Instruments). Antibodies against CD81 (1:200), CD63 (1:200), and CD9 (1:200) were used to identify the exosomes.

Exosome-packaged RNA and protein extraction was performed using a Total Exosome RNA and Protein Isolation Kit (4478545, Invitrogen). For qRT-PCR, 1.8 × 10^8^ *λ* poly A^+^ RNA was added to the exosome suspension, and exosomal lncRNAs were normalized against exogenous *λ* poly A [19] (External Standard Kit, 3789, Takara).

For the visualization of exosome internalization, exosomes isolated from CAFs were labeled with DiO (V22886, Invitrogen) and washed through exosome spin columns (MW3000, Invitrogen) to remove excess dye. DiO-labeled exosomes were added to Dil-labeled LUAD cells and visualized by laser scanning confocal microscopy (LSM710, Zeiss) after 24 h.

Exosome-packaged RNA labeling was performed using a Click-iT RNA Imaging Kit according to the manufacturer’s instructions (C10329, Invitrogen). Briefly, CAFs were labeled with 100 μM 5-EU for 24 h, and LUAD cells were labeled with Dil for 10 min. Both cells types were then washed and co-cultured for 8 or 24 h. EU was then visualized by Alexa Fluor 488 azide (Alexa Fluor 488 5-carboxamido-(6-azidohexanyl), bis(triethylammonium salt)). For 4sU RNA transfer, 4sU (4-thiouracil)-labeled CAFs were washed, left in mono-culture for 24 h, and conditioned medium collected from 4sU-labeled CAFs was added to LUAD cells. LUAD cells were then harvested 24 h later, followed by RNA extraction. 4sU-labeled RNA was biotinylated using EZ-Link HPDP-Biotin (Thermo Fisher) for 2.5 h at room temperature. Free biotin was depleted by phenol–chloroform RNA isolation, and CAF-derived 4sU-labeled RNA was enriched with Dynabeads MyOne Streptavidin C1 magnetic beads (Thermo Fisher) following the manufacturer’s instructions. CAF-derived 4sU-enriched RNA was then eluted with 1,4-dithiothreitol (Sigma) and extracted for qRT-PCR analysis.

For exosome depletion, antibodies against CD81 (1:100; Novus) and mouse IgG (1:100) were labeled with biotin using a Zenon Biotin-XX Mouse IgG2b Labeling Kit (Z25252, Molecular Probes). CAF culture medium was incubated with the biotin-labeled antibody against CD81 or IgG overnight at 4 °C, and the medium was incubated with Dynabeads MyOne Streptavidin T1 (65601, Invitrogen) for 30 min at 25 °C. The exosome-antibody complex was obtained using a magnet. GW4869, an exosome inhibitor, was also used to block exosome secretion.

### FISH and immunofluorescence staining

The quantification of LINC01614 in fibroblasts of clinical samples was determined by co-expression analysis of LINC01614 and α-SMA positive cells. Briefly, paraffin-embedded sections were deparaffinized and incubated with anti-α-SMA antibody (1:200, CST) and then incubated with Alexa Fluor 555-conjugated secondary antibody (1:10000, A32727, Thermo Fisher). The sections were dehydrated with ethanol and rehydrated in 50% formamide and then hybridized with a 20 nM 5′-digoxigenin-labeled LINC01614 probe (Ribobio) and anti-digoxigenin-FITC. DAPI was used for counterstaining the nuclei, and images were observed with laser scanning confocal microscopy (LSM710, Zeiss).

The combination of FISH and immunofluorescence experiments were used to detect the expression of LINC01614 and the interaction of proteins in CAF exosome-treated LUAD cells. Briefly, cells were fixed with 4% paraformaldehyde for 10 min at room temperature, washed with PBS, and permeabilized with 0.2% Triton-X-100 for 15 min. Cells were then blocked with goat serum for 1 h at room temperature. Thereafter, cells were incubated with primary antibodies for anti-p65 (1:200) and anti-ANXA2 (1:100) overnight at 4 °C and incubated with Alexa Fluor-conjugated secondary antibodies (Thermo Fisher) for 1 h at room temperature. The cells were then hybridized with a 5’-digoxigenin-labeled LINC01614 probe (Ribobio) and anti-digoxigenin-FITC. DAPI was used for counterstaining the nuclei, and images were captured by laser scanning confocal microscopy (LSM710, Zeiss).

### Immunohistochemistry and in situ hybridization and data analyses

Paraffin-embedded tissues were sliced at 4 μm thickness. Antigen retrieval was applied using a pressure cooker for 3 min in 0.01 M citrate buffer (pH 6.0). The sections were incubated with antibodies specific for α-SMA (1:200; CST), SLC38A2 (1:200; CST), SLC7A5 (1:200; CST), and Ki67 (1:100; CST) overnight at 4 °C, and the immunodetection was conducted on the following day with DAB. For in situ hybridization, the samples were preincubated with a hybridization solution for 2 h at 50 °C. For hybridization, an anti-LINC01614 oligodeoxynucleotide probe conjugated with DIG (Exiqon) was used. After washing, the sections were incubated with hydrogen peroxide for 15 min at room temperature and HRP-conjugated secondary antibody for 1 h at room temperature and stained with DAB, mounted, and examined.

LINC01614 was abundantly expressed in fibroblasts. The staining intensity was graded using the following scale: (0) negative; (1) low positive; (2) positive; (3) high positive. The proportion of positively stained cells in slides was determined as follows: (0) no positive cells; (1) < 25%; (2) 25–50%; (3) 50–70%; and (4) > 75%. The IHC scores were calculated by multiplying the staining intensity score and percentage of positive cells.

### qRT-PCR assay

qRT-PCR was performed using ChamQ Universal SYBR qPCR Master Mix (Q711-02/03, Vazyme) according to the manufacturer’s instructions. The primer sequences are listed in Additional file 1: Table S3. Data were collected using an Applied Biosystem Prism 7500 Fast Sequence Detection System (Applied Biosystems).

### Cytosolic/nuclear fractionation

The subcellular localization of LINC01614 was determined using the PARIS Kit (Ambion, Life Technologies, Carlsbad, CA, USA) according to the manufacturer’s instructions. Briefly, cells were incubated in cell fractionation buffer on ice for 5 min. After centrifugation at 500×*g* for 5 min, the supernatant was collected as the cytosolic fraction. The nuclear pellet was resuspended in a cell disruption buffer and incubated at 4 °C for 30 min. The nuclear fraction was obtained after removing insoluble membrane debris by centrifugation for 10 min at 12,000×*g*.

### Western blotting

Protein extracted from the cells was electrophoresed by SDS–polyacrylamide gels and was transferred to polyvinylidene difluoride (PVDF) membranes. Primary antibodies against p65 (1:1000, CST), phospho-p65 (ser 536) (1:1000, CST), phospho-p65 (ser 276) (1:1000, CST), ANXA2 (1:1000, CST), SLC38A2, SLC7A5, IKKβ, phospho-IKKβ, IKBα, phospho-IKBα, Histone 3, CD81, CD9, CD63, GAPDH, and β-actin were used. Goat anti-rabbit IgG H&L (IRDye ®800CW) preadsorbed (1:10,000, ab216773, Abcam) or Goat anti-Mouse IgG H&L (IRDye ®680RD) preadsorbed (1:10,000, ab216776, Abcam) secondary antibodies were used, and the antigen–antibody reaction was visualized using an Odyssey infrared scanner (Li-Cor, Lincoln, NE, USA).

### RACE (rapid implication of cDNA ends)

5′-RACE, 3′-RACE, and full-length amplification of LINC01614 were conducted using a SMART RACE cDNA Amplification Kit (Clontech) according to the manufacturer’s instructions.

### Transfection and transduction of tumor cells

For siRNA transfection, cells were transfected with specific siRNA duplexes using Lipofectamine 3000 (Invitrogen) following the manufacturer’s instructions. For transduction, tumor cells incubated in 24-well plates were transduced by lentiviral particles (MOI of 10) with 5 μg mL^−1^ polybrene. The oligonucleotide sequences of siRNAs and shRNAs are shown in Additional file 1: Table S2.

### Immunoprecipitation

Cells were lysed in IP lysis buffer containing protease inhibitors. The lysates were collected and centrifuged at 13,000 × *g* for 10 min, and the supernatants were transferred into new tubes for immunoprecipitation. For immunoprecipitation, an antibody against p65 (1:100, 8242, CST) was added to the lysates and incubation overnight at 4 °C, rabbit IgG (1:100) was used as the control. Dynabeads Protein A/G (10002D/10003D, Invitrogen) was then added to the tubes and incubation for 1 h at 4 °C. After washing with the lysis buffer, the immunocomplexes were resuspended in protein loading buffer and used for immunoblotting.

### RNA immunoprecipitation and pull-down assays

RNA immunoprecipitation was conducted using the Magna RIP RNA-Binding Protein Immunoprecipitation Kit (17-700, Millipore) following the manufacturer’s instructions. RNA pull-down assays were performed as previously described [20]. Briefly, biotin-labeled RNAs were transcribed using a MEGAscript T7 High Yield Transcription Kit (Invitrogen) in vitro. Bio-16-UTP was used for the in vitro transcription. To form proper secondary structure, 1 μg biotinylated RNA incubated in RNA structure buffer (10 mM Tris (pH 7), 0.1 M KCl, 20 mM MgCl_2_) was heated to 95 °C for 2 min, cooled on ice for 3 min, and incubated for 30 min at room temperature. Cell lysates were prepared by ultrasonication in RIP buffer. In vitro-transcribed and folded RNA was mixed with cell lysates at room temperature for 1 h. A total of 50 μL of washed streptavidin magnetic beads (60,210, Invitrogen) was added to the binding reaction and then incubated at 4 °C for 4 h before washing with RIP buffer five times and elution in Laemmli sample buffer. SDS-PAGE then separated the retrieved proteins for MS or western blotting.

### In vitro binding assay

GST-conjugated p65 (ab114150, Abcam) and His-conjugated ANXA2 (ab93005, Abcam) were used. In vitro binding assays were conducted in IP lysis buffer. GST-conjugated p65, His-conjugated ANXA2, and folded LINC01614 were incubated at room temperature for 1 h. The interaction was determined by immunoprecipitation and western blotting.

### Chromatin immunoprecipitation assays

Chromatin immunoprecipitation assays were conducted using an EZ-Magna ChIP chromatin immunoprecipitation kit (17-371RF, Millipore) according to the manufacturer’s instructions. Briefly, after fixation in 1% formaldehyde for 10 min at room temperature, 5 × 10^6^ cells were collected, lysed, and sonicated. Antibodies against p65 (5 μL mg^−1^ protein) and rabbit IgG (2 μL mg^−1^ protein) were used for immunoprecipitation. Bound DNA fragments were subjected to real-time PCR. The primer sequences used in the ChIP assays are provided in Additional file 1: Table S3.

### Luciferase reporter assays

Luciferase reporter assays were conducted according to the manufacturer’s instructions (Promega). pGL3-based constructs containing the wild-type (WT) or MUT SLC38A2 and SLC7A5 promoters together with the *Renilla* luciferase plasmids were transfected into cells using Lipofectamine 3000 (Invitrogen). Luciferase activity was examined 48 h after transfection by the Dual-Luciferase Reporter Assay System, and firefly luciferase activity was normalized to *Renilla* activity.

### Flow cytometry

Suspended cells were stained with Live/Dead Fixable Viability Dye (FVD-eFluor780, 65-0863-14, eBioscience) in PBS for 20 min to distinguish the live cells from dead cells. For cell surface marker analysis, cells resuspended in PBS containing 1% GBS were stained with fluorescent-conjugated antibodies against CD326 (EpCAM), CD140b (PDGFR-β), CD45, and fibroblast-specific protein (FSP) for 30 min at 4 °C according to the manufacturer’s instructions. The following monoclonal antibodies were used: human CD140b (323605, BioLegend), human fibroblast (130-100-133, Miltenyi), human CD326 (130–111-118, Miltenyi), and human CD45 (304019, BioLegend). Samples were analyzed using a BD Accuri C6 Flow Cytometer. For gating myofibroblasts (myCAF), inflammatory CAF (iCAF) and antigen-presenting CAF (apCAF), antibodies against I-A/I-E (BioLegend, MHC class II), Ly6C (BioLegend), IL-6 (BioLegend) and α-SMA were used: FSP^+^MHCII^+^ (apCAFs), FSP^+^MHCII^−^IL6^+^ (iCAFs), FSP^+^MHCII^−^α-SMA^+^ (myCAFs).

### ELISA

LUAD cells were cultured with DMEM containing 10% FBS until 80% of confluency. The cells were cultured in fresh serum-free media after washing with PBS. After 24 h, the supernatants were collected and used for ELISA assay. The IL-6, CXCL10, and CCL5 ELISA kits were purchased from Fcmacs. All assays were performed according to the manufacturer’s instructions.

### Non-tumor cell-expressed lncRNAs

First, we used TCGA datasets to identify LUAD upregulated lncRNAs. RNA sequencing (RNA-Seq) data from 585 LUAD cases were downloaded from the data portal (https://portal.gdc.cancer.gov), including 56 normal lung tissue samples. The R package DESeq was used to count data [21], and 7320 differentially expressed genes were detected (fold change > 2 and FDR < 0.05) among 60,483 genes. According to the "Gene_type" annotation by the Ensembl genes database (Hg38), 649 lncRNAs were screened from differentially expressed genes as upregulated lncRNAs. Second, to reveal potential non-tumor cell-expressed lncRNAs, we considered the expression levels of all 649 upregulated lncRNAs in 57 LUAD cell lines and the normal epithelial line SALE. We performed the DESeq method between every LUAD cell line and SALE, and we found that a total of 195 lncRNAs could not be detected as highly expressed in any LUAD cell lines (*P-*value < 0.1). Third, to assess the association between TME compositions and non-tumor cell-expressed lncRNAs, we applied the MCP-counter tool to calculate abundance scores of TME populations [22], and we performed correlation analysis using the Pearson’s correlation coefficient.

### GSEA on RNA sequencing

Total RNA of fibroblasts, LUAD cells, and educated LUAD cells was extracted with TRIzol. Exosome-packaged RNA extraction was performed using Total Exosome RNA and Protein Isolation Kit (4478545, Invitrogen). For RNA sequencing, libraries were prepared from purified RNA using a NEBNext Ultra Directional RNA Library Prep Kit to ensure the RNA was not fragmented prior to library preparation. Libraries were then sequenced on Illumina HiSeq 2500 with 100 base paired-end reads. HISAT, StringTie, and Ballgown analysis were used as previously described [23]. For Ballgown, we used transcript FPKM as the transcript expression measurement, upon which we performed GSEA between experimental groups [24].

### Subcutaneous tumorigenicity and tail vein injection assay

For the subcutaneous tumorigenicity assay, 4-week-old female BALB/c nude mice were purchased from Vital River Laboratories and maintained under standard conditions according to protocols approved by the Nanjing Medical Experimental Animal Care Commission (approval number: IACUC-2101034). Tumor cells (5 × 10^6^ A549 cells) were subcutaneously injected into one flank of each mouse in 0.1 mL of sterile PBS. After the inoculation, 100 μL of PBS containing exosomes obtained from CAFs with indicated treatment (0.5 μg kg^−1^) was injected peritumorally every 3 d. For the co-injection mouse model, 5 × 10^6^ A549 cells and 5 × 10^6^ transduced CAFs were co-injected into a single flank of each mouse in 0.1 mL of sterile PBS.

For the tail vein injection assay, 5 × 10^6^ luciferase-labeled A549 cells were intravenously injected into female NCG mice (NODprkdc^−/−^IL-2Rg^−/−^) through the tail vein (GemPharmatech, Nanjing, China). Two weeks after injection, mice were randomly divided into groups and intravenously injected with an equal number of exosomes obtained from transduced CAFs once a week for 4 weeks. After another week, lung metastasis was assessed and quantified by ex vivo bioluminescent imaging using IVIS Lumina Series III (PerkinElmer, USA).

### Zebrafish tumor model

Zebrafish embryos of the transgenic strain expressing enhanced GFP with the fli1 promoter (Fli1: EGFR) [25] were incubated at 28 °C under standard experimental conditions according to protocols approved by the Nanjing Medical Experimental Animal Care Commission. At 48 hpf, Fli1:EGFR zebrafish larvae were anesthetized with 0.04 mg mL^−1^ tricaine (MS-222, Sigma). Anesthetized larvae were subjected to microinjection. LUAD tumor cells and CAFs were labeled with 2 μg mL^−1^ DiD (V-22887, Chroma) or CM-Dil (V-22888, Chroma), respectively. A total of 500 labeled LUAD cells or a mixture containing 300 LUAD cells and 200 CAFs were resuspended in DMEM, and 5 nL of the cell solution was injected into the perivitelline space (PVS) of larvae [25]. For exosome treatment, CAF-derived exosomes (5 ng) were injected into the PVS of larvae 24 h after cells implantation [26]. After injection, the larvae were immediately transferred into the aquarium water supplemented with 0.2 mmol L^−1^ 1-pheny-2-thio-urea (P7629, Sigma). Four days after injection, the zebrafish were fixed and analyzed for tumor invasion and metastasis using laser scanning confocal microscopy (LSM710, Zeiss).

### Statistics

Data were analyzed with GraphPad Prism, SPSS 20 software, or R programming language. For most in vitro and animal experiments, Student’s two-tailed *t*-tests were used for single comparisons (paired or unpaired), and a one-way ANOVA was performed for multiple comparisons. Spearman order correlation analysis was used to determine the relationship between different factors. Survival curves were plotted using the Kaplan–Meier method and assessed using log-rank tests. All experiments were repeated independently three times. Animal studies were repeated using five to eight independent mice per group. Data are shown as the mean ± standard deviation (s.d.) unless stated otherwise. The *P*-value < 0.05 was considered to be statistically significant.

## Results

### Exosome-packaged RNA from CAFs enhances glutamine uptake and progression of LUAD cells

Given the increasing evidence supporting the cancer-promoting effects of CAFs [27], we first confirmed the contribution of CAFs in LUAD. We studied the number of activated fibroblasts identified by α-SMA immunohistochemical (IHC) staining in a tissue microarray (TMA) of LUAD (Additional file 2: Fig. S1A). α-SMA is highly overexpressed in LUAD (compared with normal lung tissue) (Additional file 2: Fig. S1B). α-SMA^+^ CAF density is positively correlated with the T stage (Additional file 2: Fig. S1C). High expression of α-SMA is associated with poor overall survival (HR = 2.791, 95% CI 0.1839–0.6982; *P* = 0.0017) (Additional file 2: Fig. S1D). Functional experiments in vitro were performed using primary CAFs and paired normal fibroblasts (NFs) derived from human LUAD samples (Additional file 2: Fig. S1E-F). We established a co-culture Transwell system in a non-interacting manner, as previously reported [11]. We found that LUAD cell lines (glutamine-sensitive A549 and glutamine-insensitive H1975, respectively) co-cultured with CAFs exhibited higher proliferation, migration, and invasion abilities (Additional file 2: Fig. S1G-I) than those cultured alone or co-cultured with NFs.

**Fig. 1:**
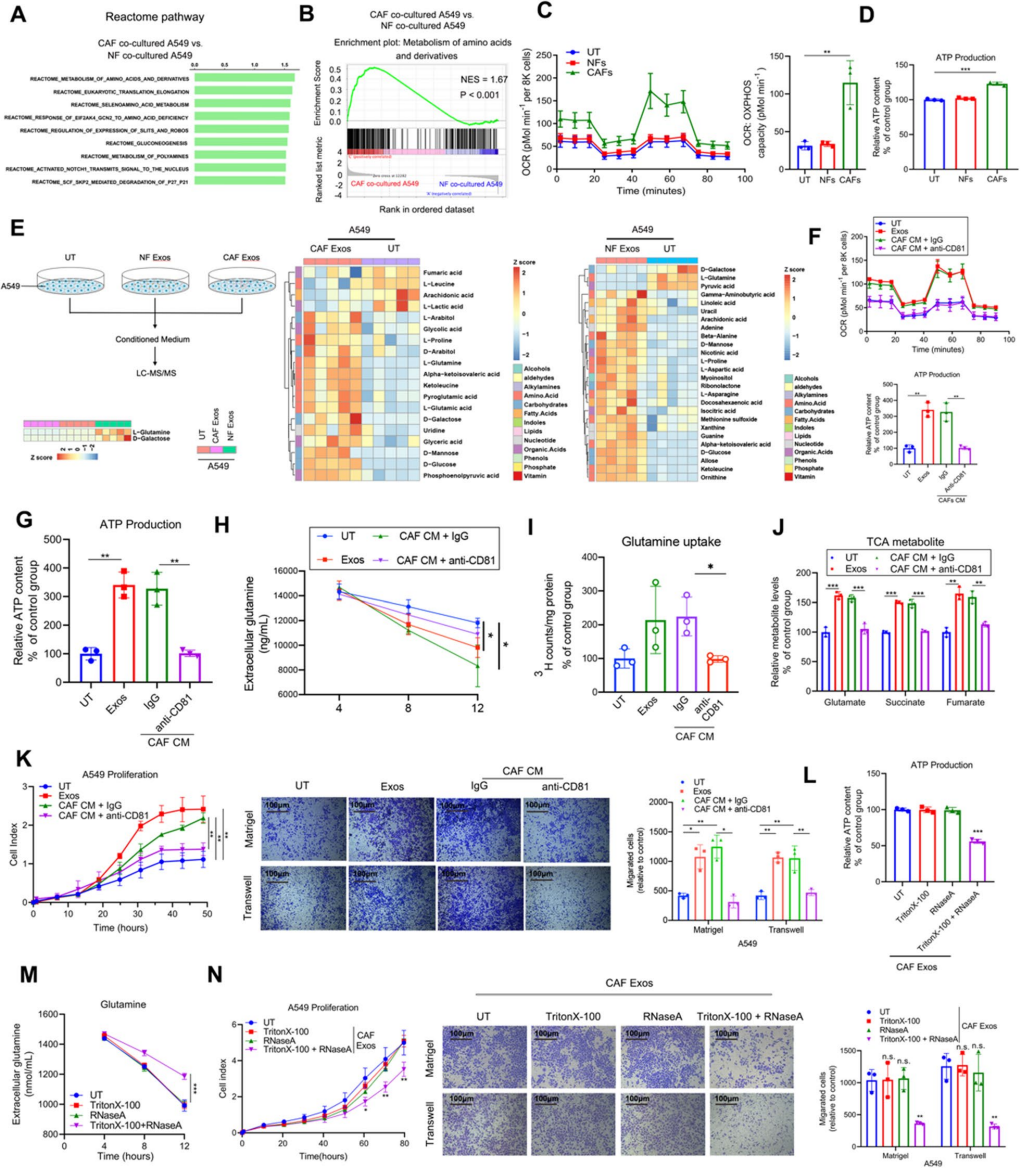
Exosome-packaged RNA from CAFs enhances the glutamine uptake and progression of LUAD cells. **A**, **B** Representative transcriptome analysis of the Reactome pathway (**A**) and GSEA (**B**) in the A549 cells co-cultured with CAFs compared with those with NFs (*n* = 3). **B** Reactome pathway-based GSEA revealed an enrichment of “Metabolism of amino acids and derivatives” in the A549 cells co-cultured with CAFs compared to NFs co-cultured with A549 cells (*n* = 3). **C** OCR of A549 cells with indicated treatments (*n* = 3). **D** ATP production of A549 cells with indicated treatments (*n* = 3). **E** Schematic and heatmap of metabolomic experiments for conditioned media of indicated cells. **F**–**L** A549 cells were treated with exosomes or CM of CAFs and then used for the indicated experiments. anti-CD81 antibody was used for depleting exosomes in the CM of CAFs. **F** OCR of A549 cells with the indicated treatments (*n* = 3). **G** ATP production of A549 cells with indicated treatments (*n* = 3). **H** Extracellular glutamine levels of A549 cells cultured for 4, 8, and 12 h in 25 mM glucose DMEM (containing 1 mM pyruvate and 4 mM glutamine) with the indicated treatments (*n* = 3). **I** ^3^H-glutamine uptake (*n* = 3). **J** Glutamine-derived TCA cycle intermediates in A549 cells with indicated treatments (*n* = 3). **K**, **L** RTCA proliferation, migration (lower), and invasion (upper) assays and ATP production of A549 cells with indicated treatments. **M** Extracellular levels of glutamine of A549 cells with the indicated treatments (*n* = 3). **N** RTCA proliferation assays, migration (lower), and invasion (upper) assays of A549 cells with indicated treatments (*n* = 3). For **C**, **D** and **F–N**, Means ± s.d. are shown, and independent sample *t-*tests determined P values. * *P* < 0.05, ** *P* < 0.01, *** *P* < 0.001. UT, cancer cells without any treatment; CM, conditioned medium; Exos, exosomes; GSEA, gene set enrichment analysis; RTCA, Real-time xCELLigence analysis; LUAD, lung adenocarcinoma

To further identify the molecular mechanisms underlying CAF-mediated progression in LUAD, we performed RNA-seq analysis to compare the RNA expression profiles in A549 cells cultured alone or co-cultured with three pairs of NFs and CAFs. (LUAD01, LUAD02 and LUAD03 patients-derived CAFs and NFs were used here.) Reactome pathway analysis and gene set enrichment analysis (GSEA) for transcriptomes showed that genes involved in the metabolism of amino acids and derivatives were remarkably upregulated in A549 cells co-cultured with CAFs (Fig. 1A, B). Moreover, GESA based on KEGG pathways revealed that amino acid metabolism—primarily alanine, aspartate, and glutamine—was elevated in A549 cells co-cultured with CAFs (Additional file 2: Fig. S1J). To investigate whether CAFs contribute to the metabolism of LUAD cells, we examined changes in the mitochondrial oxygen consumption rate (OCR) and extracellular acidification rate (ECAR), measures of mitochondrial activity and glycolysis, respectively, in A549 cultured alone or co-cultured with NFs and CAFs (Fig. 1C; Additional file 2: Fig. S1K). A549 cells showed few changes in ECARs, glucose consumption, or lactate production when co-cultured with NFs or CAFs (Additional file 2: Fig. S1K-L). In contrast, LUAD cells co-cultured with CAFs exhibited higher mitochondrial OCRs (Fig. 1C) and ATP synthesis (Fig. 1D).

Given the metabolic changes in A549 cells following co-culture with CAFs, we hypothesized that mediators in the supernatants might be responsible. Because exosomes play important roles in the communication between cells [28], we next performed a series of metabolomic studies to identify the differentially expressed metabolites in the conditioned media (CM) of A549 treated with indicated exosomes (Fig. 1E). Notably, we observed that glutamine was the top metabolite under-represented in the CM of A549 cells co-cultured with CAF exosomes. Because glutamine is an energy source for cancer cells, we determined the cellular uptake of ^3^H-glutamine in A549 cells. A549 cells co-cultured with CAFs exhibited higher glutamine uptake than those cultured alone or co-cultured with NFs. Additionally, A549 cells co-cultured with CAFs had a higher content of TCA cycle intermediates (Additional file 2: Fig. S1M). Similarly, we observed enhanced glutamine consumption rate and ATP production in H1975 cells co-cultured with CAFs (Additional file 2: Fig. S1N). These data suggest that CAFs increase the uptake and utilization of glutamine in LUAD cells in vitro in a non-interacting manner.

Next, we determined whether CAF-derived exosomes mediated the uptake of glutamine and the progression of LUAD cells. Exosomes in CAFs CM were isolated by ultracentrifugation. The morphology, diameter, and biomarkers of the exosomes were confirmed by electron microscopy, NanoSight analysis, and western blotting (Additional file 2: Fig. S2A-D), respectively. The internalization of CAF-derived exosome cargo by the tumor cells was visualized by confocal microscopy (Additional file 2: Fig. S2E). CAF-derived exosomes remarkably enhanced the mitochondrial OCR, glutamine consumption rate, glutamine influx, ATP synthesis, and abundance of TCA intermediates derived from glutamine catabolism (Fig. 1F–J; Additional file 2: Fig. S2F-G), and the proliferation, migration, and invasion of LUAD cells (Fig. 1K; Additional file 2: Fig. S2H). More importantly, depleting exosomes from CAFs CM using an anti-CD81 antibody significantly inhibited their roles in promoting glutamine metabolism and proliferation, migration, and invasion of LUAD cells (Fig. 1F–K; Additional file 2: Fig. S2F-H). Collectively, these results demonstrated that CAF-derived exosomes enhanced glutamine influx and promoted the progression of LUAD cells.

Recent studies have shown that exosomal RNAs are critical in the cellular communication between tumor cells and the TME [11]. We next investigated whether CAF-derived exosomal RNAs mediated the enhancement of glutamine influx and LUAD progression. We observed that pretreated exosomes with RNase and Triton-X-100 abolished the effects of CAFs on LUAD cells (Fig. 1L–N). These data further confirmed that exosome-packaged RNAs from CAFs at least partially contribute to enhancing glutamine metabolism in LUAD**.**

### Validation and transmission of CAF-specific lncRNA LINC01614

**Fig. 2:**
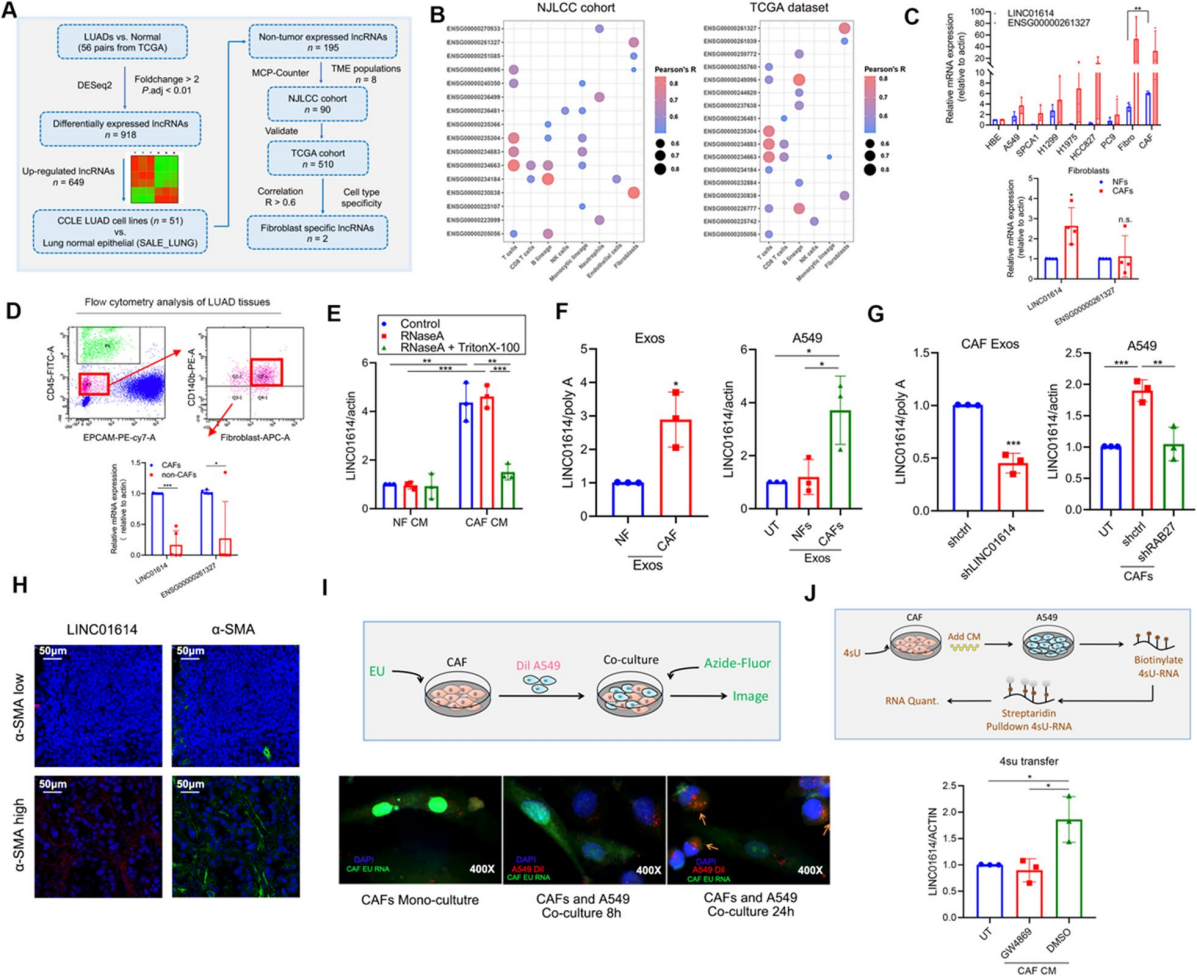
Intercellular transfer of a CAF-specific lncRNA LINC01614 by exosomes. **A** Schematic showing the screening strategy of TME-specific lncRNAs. **B** LncRNAs that are moderately to highly positively correlated with TME cells in the NJLCC cohort (left) and TCGA database (right) (*ENSG0000230838* is for LINC01614). **C** qRT-PCR of LINC01614 (*ENSG0000230838*) and *ENSG00000261327* in CAFs, NFs, MRC5, and LUAD cell lines (*n* = 3). **D** Flow cytometric sorting of CAFs from cell suspension of human LUAD tissue. Live/Dead Fixable Viability Dye staining was used to detect dead cells. CD45 was used as an immune cell marker, EpCAM as an epithelial cell marker, fibroblast-specific protein (FSP) and PDGFR β (CD140b) as fibroblast markers. qRT-PCR analysis of LINC01614 and ENSG00000261327 in isolated CAFs and CAF-depleted cells (*n* = 3). **E** qRT-PCR of LINC01614 and ENSG00000261327 in the CM of A549 cells treated with RNase (2 μg mL^−1^) alone or combined with Triton-X-100 (0.1%) for 20 min (*n* = 3). **F** qRT-PCR analysis of LINC01614 in CAFs exosomes versus NFs exosomes (*n* = 3), and A549 cells with indicated treatments versus those cultured alone for 24 h (*n* = 3). **G** Relative expressions of LINC01614 in CAF-derived exosomes (left) and the A549 cells co-cultured with the CAFs transfected with shRAB27 (right) were determined by qRT-PCR. **H** Representative images of α-SMA immunofluorescent staining and LINC01614 fluorescence in situ hybridization staining in LUAD patient tissues (*n* = 10). **I** Schema and representative images for measuring RNA transfer from CAFs to A549 cells utilizing the uridine analog EU for fluorescence microscopy (green). CAFs were labeled with EU and co-cultured with Dil lipid-labeled A549 cells for the indicated time. Representative images of EU-positive A549 cells (orange) are shown (*n* = 3). **J** Schema for measuring LINC01614 transfer from CAFs to A549 cells. Conditioned medium from CAFs labeled with 4sU was added to A549 cells, 4sU RNA in A549 cells was isolated with streptavidin pull-down. Relative transfer of 4sU RNA to mono-cultured A549 cells the addition of CM collected from 4sU-labeled CAFs. CAFs CM depleted of exosomes with GW4869 is shown as a control for exosome-dependency (*n* = 3). For all experiments, means ± s.d. are shown, and independent sample *t-*tests determined P values. *P < 0.05, **P < 0.01, ***P < 0.001. *TME* tumor microenvironment; *NJLCC* Nanjing lung cancer cohort; *UT* cancer cells without any treatment; *CM* conditioned medium; Exos, exosomes; *EU* 5-ethynyl uridine; *4sU* 4-thiouridine; *LUAD* lung adenocarcinoma. Source data are provided

lncRNAs have recently exhibited cell lineage- and developmental stage-dependent expression patterns [29]. Lineage-specific and cell-specific expressed lncRNAs control lineage-specific regulatory programs and regulate pathophysiological processes, including cancer [11, 29, 30]. To determine whether CAF-specific lncRNAs mediate the glutamine addiction of LUAD cells, we screened CAF-specific lncRNAs based on the NJLCC cohort (https://ega-archive.org/datasets/EGAD00001004071), who were recruited in Jiangsu Cancer Hospital. A total of 149 patients with non-small cell lung cancer were enrolled, and we examined TCGA data and their presence in exosomes. To screen CAF-associated lncRNAs, we first performed an analysis to identify overexpressed lncRNAs in LUAD tissues based on TCGA LUAD datasets. A total of 649 lncRNAs were upregulated in LUAD tissues (Fig. 2A; Additional file 2: Fig. S3A). To reveal potential non-tumor cell-expressed lncRNAs, we analyzed the expression levels of the 649 upregulated lncRNAs in 57 lung LUAD cell lines and the normal epithelial line SALE based on the Cancer Cell Line Encyclopedia (CCLE) database. Interestingly, 195 lncRNAs were not highly expressed in any LUAD cell lines, and they were identified as the TME-associated lncRNAs (Additional file 2: Fig. S3B). Furthermore, we performed correlation analysis to determine the correlation of the 195 lncRNAs with TME populations based on the NJLCC cohort and TCGA LUAD datasets. Among the 195 TME-associated lncRNAs, 20 lncRNAs were highly positively correlated with 20 types of LUAD stromal cells, including two highly positively CAFs-associated lncRNAs (LINC01614 (*ENSG000000230838*) and *ENSG00000261327*) (Fig. 2B). TCGA data showed that LINC01614 and *ENSG00000261327* were highly upregulated in several cancers, including LUAD (Additional file 2: Fig. S3C). Moreover, the Genotype-Tissue Expression (GTEx) dataset showed that LINC01614 was specifically expressed in cell-cultured fibroblasts, whereas *ENSG00000261327* was relatively upregulated in cell-cultured fibroblasts, (Additional file 2: Fig. S3D-E).

Quantitative reverse transcription PCR (qRT-PCR) confirmed that LINC01614 rather than *ENSG00000261327* was highly expressed in CAFs compared with LUAD cell lines, MRC5 cells (human embryonic lung fibroblasts), and paired NFs (Fig. 2C). Moreover, we isolated CAFs from LUAD tissues by fluorescence-activated cell sorting (FACS), and LINC01614—rather than *ENSG00000261327*—was primarily expressed in CAFs and not in non-CAF cells (Fig. 2D). Given that CAF exosomal RNA enhanced glutamine influx and the progression of LUAD, we investigated whether LINC01614 could be released from CAFs by exosomes. The expression of LINC01614 in CAF CM was unchanged upon RNase treatment but significantly decreased when simultaneously incubated with RNase and Triton-X-100 (Fig. 2E), suggesting that LINC01614 could be released from CAFs and the extracellular LINC01614 was primarily wrapped by membranes instead of being directly released. Furthermore, LINC01614 was increased more than fivefold in the exosomes from CAFs compared to NFs and by more than threefold in A549 cells co-cultured with CAF-derived exosomes (Fig. 2F). qRT-PCR results showed that LINC01614 was highly expressed in myofibroblasts (myCAF) as compared to inflammatory CAF (iCAF) and antigen-presenting CAF (apCAF). We proposed myCAF subpopulation was the main source of LINC01614-containing exosomes (Additional file 2: Fig. S3F).

To confirm whether the increase in LINC01614 in CAF co-cultured A549 cells was caused by exosome transmission, we knocked down LINC01614 and RAB27, a key enzyme for exosome secretion in CAFs before co-culture, to abolish LINC01614 release and exosome secretion, respectively (Fig. 2G). Inhibition of exosome secretion also inhibited the upregulation of LINC01614 in A549 cells co-cultured with CAFs (Fig. 2G). Importantly, silencing LINC01614 in CAFs decreased the LINC01614 level in CAF exosomes, while overexpressing LINC01614 increased the LINC01614 level in CAF exosomes (Fig. 2G; Additional file 2: Fig. S3G). However, LINC01614 did not impact the general production of exosomes from CAFs (Additional file 2: Fig. S3H-I). Moreover, fluorescence in situ hybridization (FISH) and immunofluorescence (IF) analysis showed that LINC01614 was abundantly expressed in CAFs, and its expression was positively associated with the density of CAF infiltration as determined by α-SMA IF staining of LUAD tissues (Fig. 2H). To further confirm whether CAF-derived LINC01614 accompanies exosomes transfer, we metabolically labeled CAF RNA with 5-ethynyl uridine (EU) prior to co-culture with fluorescently labeled A549 cells. After 24 h, A549 cells significantly acquired CAF RNA as assessed by EU-modification with azide-linked fluorescein (Fig. 2I). Moreover, when CAFs were similarly labeled with 4-thiouridine (4sU), treatment with CAF CM led to LINC01614 transfer from CAFs to A549 cells, which was examined by a streptavidin pull-down of biotinylated 4sU-labeled CAF RNA. In contrast, LINC01614 failed to transfer to A549 cells when exosomes were depleted from the CM using the exosome inhibitor GW4869 (Fig. 2J), consistent with our findings LINC01614 transmission is exosome-dependent. Together, these data indicated that a CAF-specific lncRNA-LINC01614 could be released and transmitted from CAFs to LUAD cells by exosomes.

### CAF-exosome-packaged LINC01614 is an oncogenic lncRNA and enhances the glutamine uptake of LUAD cells via upregulation of amino acid transporters

**Fig. 3:**
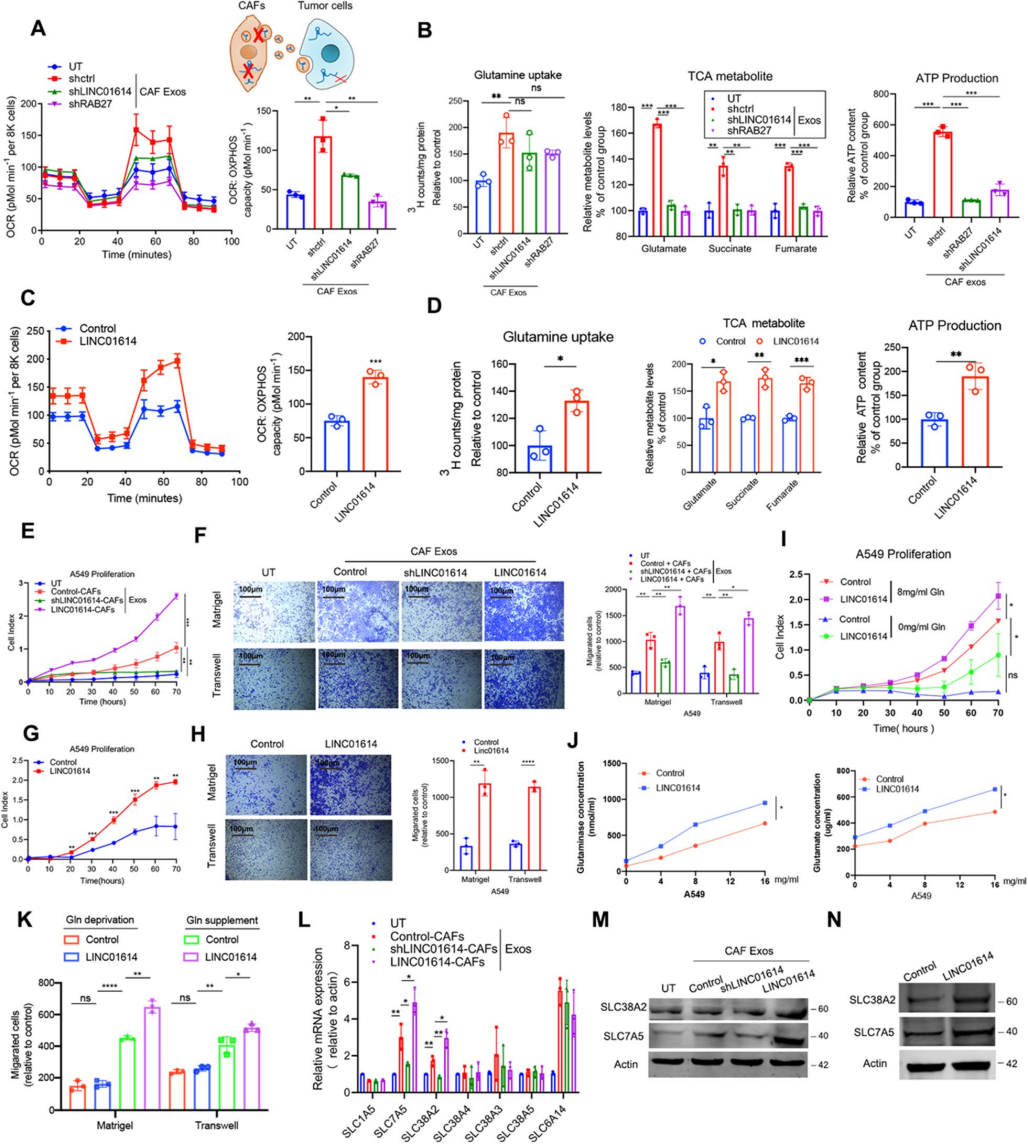
CAF-exosome-packaged LINC01614 enhances glutamine influx of LUAD cells via upregulating amino acid transporters. **A**, **B**, Exosomes isolated from the CM of CAFs transduced with lenti-LINC01614-shRNA or transfected with shRAB27 were added to A549 cells for 48 h. **A** OCR values of A549 cells were measured (*n* = 3). **B** ^3^H-glutamine uptake, glutamine-derived TCA cycle intermediates, and ATP content of A549 cells with the indicated treatments (*n* = 3). **C**, **D** and **G**, **H** A549 cells transfected with pcDNA 3.1-LINC01614. Antisense for LINC01614 was used as the control. **C**, OCR values of A549 cells (*n* = 3). **D** ^3^H-glutamine uptake, glutamine-derived TCA cycle intermediates, and ATP content of A549 cells with the indicated treatments (*n* = 3). **E–H** Quantification of the proliferation (**E** and **G**) and migration and invasion (**F** and **H**) of A549 cells with indicated treatments assessed by RTCA and Boyden chamber assay with or without Matrigel-coated inserts (*n* = 3). **I–K** RTCA proliferation, glutaminase and glutamate content, migration and invasion assays of A549 cells with indicated treatments. **L–M** Exosomes isolated from CM of CAFs transduced with lenti-LINC01614-shRNA or lenti-LINC01614 were added to A549 cells for 48 h. **L** qRT-PCR analysis was used to determine the mRNA profile of glutamine transporters in A549 cells with indicated treatments. **M–N**, Western blotting of SLC38A2 and SLC7A5 expression in A549 cells with indicated treatments. For **A–K**, means ± s.d. are shown, and independent sample *t*-tests *P* values were determined. *P < 0.05, **P < 0.01, ***P < 0.001. UT, cancer cells without treatment; CM, conditioned medium; RTCA, Real-time xCELLigence analysis; Exos, exosomes

Either silencing LINC01614 with shLINC01614 in CAFs or inhibiting exosome secretion by knocking down RAB27 abrogated the CAF-induced glutamine influx and utilization in LUAD cells (Fig. 3A, B; Additional file 2: Fig. S4A-B). Ectopic LINC01614 expression enhanced glutamine utilization in LUAD cells (Fig. 3C, D; Additional file 2: Fig. S4C-D). When treating LUAD cells, exosomes from LINC01614-silencing CAFs suppressed the enhanced proliferation, migration, and invasion, whereas the exosomes from CAFs ectopically expressing LINC01614 promoted those abilities (Fig. 3E, F; Additional file 2: Fig. S4E-F). Moreover, ectopically expressing LINC01614 also remarkably promoted the malignant behavior of LUAD cells (Fig. 3G, H; Additional file 2: Fig. S4G-H). Next, we investigated whether LINC01614-related effect on LUAD cells progression is linked to specific glutamine-addiction of cancer cells. Our results showed that both A549 and H1975 cells were increasingly addicted to glutamine as supplemented glutamine concentration increased. And ectopic LINC01614 expression enhanced the proliferation, migration and invasion behaviors of LUAD cells, however failed to enhance the malignant behaviors of LUAD cells when glutamine was deprived (Fig. 3I–K; Additional file 2: Fig. S4I-K). Moreover, CAFs showed no difference in glutamine uptake with LINC01614 overexpression or knockdown (Additional file 2: Fig. S4L). With respect to the proliferation of CAF, we found that only LINC01614 overexpression slightly enhanced CAF proliferation, while silencing LINC01614 had no effects (Additional file 2: Fig. S4M). These data suggested that modulating LINC01614 in CAFs did not impact the glutamine uptake of CAFs, while slightly affected the CAF growth. Collectively, these data further confirmed that LINC01614 facilitated LUAD cells specifically addicted to glutamine for malignant progression.

Subsequently, we hypothesized that amino acid transporters could contribute to LINC01614-mediated glutamine influx. Solute carrier families are important transporters for glutamine [31]. Therefore, we examined the transcriptional profiling of previously identified glutamine transporters in A549 cells by qRT-PCR. Only SLC38A2 and SLC7A5 were upregulated in A549 cells at mRNA and protein levels when treated with CAF exosomes. Silencing LINC01614 attenuated SLC38A2 and SLC7A5, whereas overexpressing LINC01614 enhanced their expressions in A549 cells treated with CAF exosomes (Fig. 3L, M). Moreover, ectopic LINC01614 expression in A549 cells enhanced the expressions of SLC38A2 and SLC7A5 (Fig. 3N). More importantly, both silencing SLC38A2 and SLC7A5 in A549 cells partially reversed enhanced glutamine uptake and utilization and the proliferation, migration, and invasion of ectopic LINC01614 expression in A549 cells (Additional file 2: Fig. S4N-S). Collectively, these data demonstrated that CAFs enhanced glutamine influx and utilization and the malignant behaviors of LUAD cells by transmitting a CAF-specific lncRNA-LINC01614 via exosomes.

### LINC01614 enhances ANXA2 and p65 interactions and promotes NF-κB activation

**Fig. 4:**
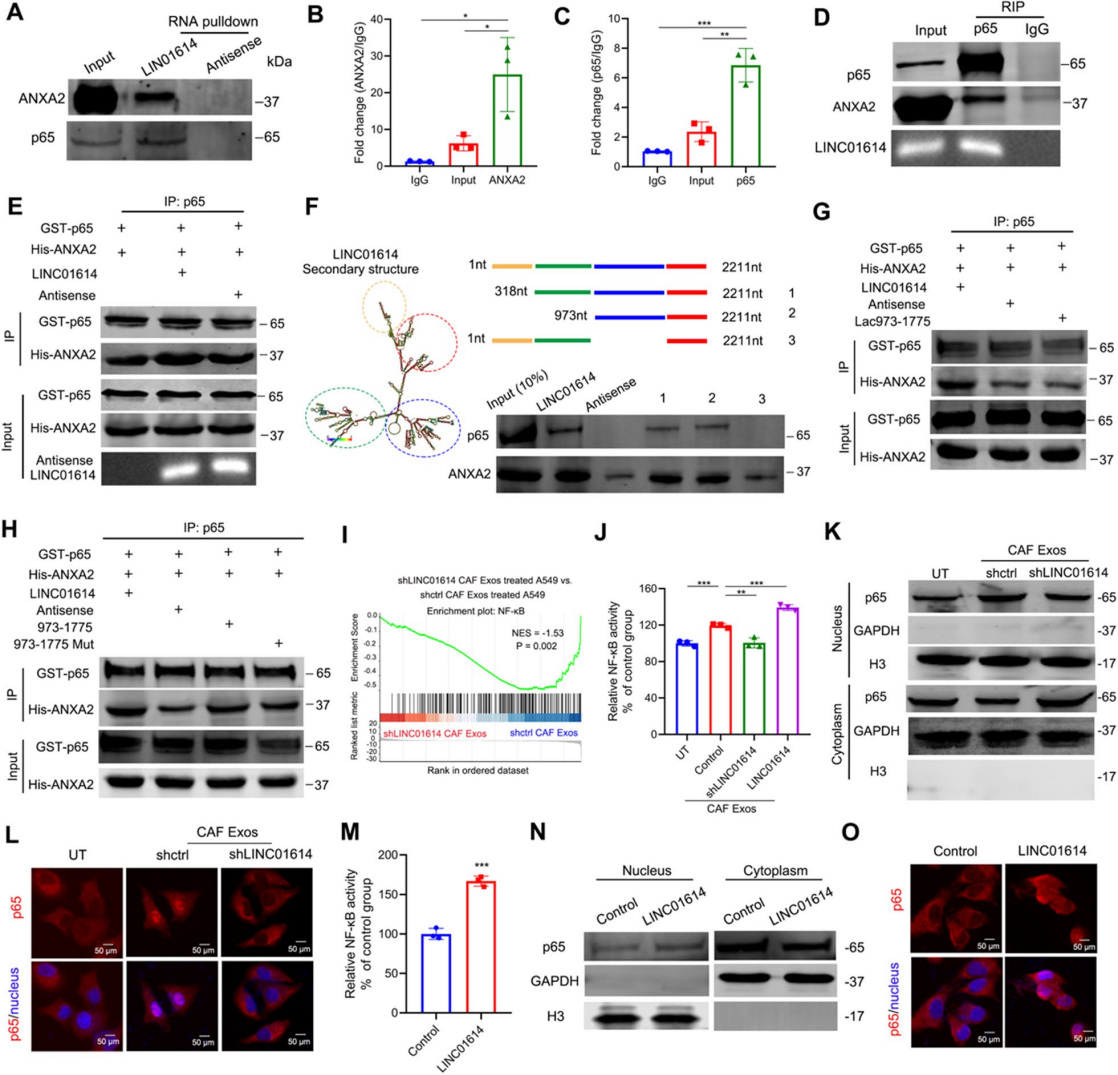
LINC01614 enhances ANXA2 and p65 interactions and promotes NF-κB activation. **A** p65 and ANXA2 were pulled down by biotin-labeled LINC01614 but not LINC01614 antisense RNA in whole-cell lysates of A549 cells treated with exosomes from CAFs (*n* = 3). **B**, **C** RIP evaluation of the interaction between ANXA2 (**B**) and p65 (**C**) using anti-ANXA2 and anti-p65 antibodies in A549 cells treated with exosomes from CAFs (*n* = 3). **D** ANXA2 and LINC01614 were co-precipitated with p65 in whole-cell lysates of A549 cells treated with exosomes from CAFs (*n* = 3). **E** IP (immunoprecipitation) analysis for the in vitro interaction of ANXA2 and p65. LINC01614 promoted the binding between recombinant ANXA2 and p65 in vitro (*n* = 3). **F** The secondary structure of LINC01614 is shown as predicated by the centroid method (http://rna.tbi.univie.ac.at). RNA pull-down assay for the interactions of sequentially deleted LINC01614 variants with ANXA2 and p65 in ectopic LINC01614 expressed A549 cells (*n* = 3). Schematic of sequentially deleted LINC01614 variants (left). Representative western blot for ANXA2 and p65 pulled down by LINC01614 variants (right). **G** nucleotide mutation lacking the stem-loop structure of LINC01614 (973–1775) abolished the interaction between p65 and ANXA2 as revealed by IP analysis. **H** LINC01614^973–1775^ enhanced the interaction between p65 and ANXA2, as revealed by IP analysis. **I** GSEA revealed enrichment of NF-κB target genes in the exosome-packaged LINC01614 treated A549 cells. **J** NF-κB activity of CAF-exosome-treated A549 cells, examined by luciferase reporter assay (*n* = 3). **K** Western blot analysis of the nuclear factor NF-κB p65 subunit following nuclear fractionation of exosome treated A549 cells. **L** Immunofluorescent p65 staining showing nuclear translocation in A549 cells with indicated treatments (*n* = 3). **M** NF-κB activity of A549 transduced with lenti-LINC01614, determined by luciferase reporter assay (*n* = 3). **N** Western blot analysis of the nuclear factor NF-κB p65 subunit following nuclear fractionation of A549 cells transduced with LINC01614. Loading controls, GAPDH (cytoplasmic fractions), and H3 (nuclear fractions) (*n* = 3). **O** Immunofluorescent p65 staining showing its nuclear translocation in A549 cells with indicated treatments (*n* = 3). For **B**, **C**, **J**, and **M** means ± s.d. are shown, and independent sample *t*-tests determined *P* values. *P < 0.05, **P < 0.01, ***P < 0.001. *UT* cancer cells without any treatment; *CM* conditioned medium; *Exos* exosomes; *GESA* gene set enrichment analysis; *LUAD* lung adenocarcinoma

To address how LINC01614 promotes glutamine uptake, we first performed nuclear mass separation and FISH assays in CAFs. Our results showed that LINC01614 was mainly distributed in the CAF cytoplasm (Additional file 2: Fig. S5A-C), consistent with the supposition that CAF-derived exosomes could package LINC01614. Next, we used an RNA pull-down in vitro-transcribed LINC01614 RNA with a biotin-labeled 3ʹ end in A549 cells and analyzed the protein products with mass spectrometry (MS). The p65 (Rela) and Annexin A2 (ANXA2) were associated with LINC01614 (Additional file 2: Fig. S5D-E). This was confirmed by immunoblotting and RNA immunoprecipitation (RIP) assays (Fig. 4A–C). Moreover, LINC01614 along with ANXA2 could be co-immunoprecipitated with p65 (Fig. 4D), suggesting that LINC0164, ANXA2, and p65 could form a ternary complex. To confirm this, we used an ex vivo system with recombinant proteins (p65 and ANXA2) and in vitro-transcribed LINC01614 as previously described [11]. The addition of LINC01614, but not antisense lncRNA, effectively enhanced the interaction between p65 and ANXA2 (Fig. 4E). To further investigate the segments of LINC01614 that associate with p65 and ANXA2, we generated a series of truncated versions of LINC01614. Nucleotides 973–1775 of LINC01614 interacted with p65 and ANXA2 (Fig. 4F). Moreover, a nucleotide mutation lacking the stem-loop structure of LINC01614 (973–1775) attenuated the LINC01614-enhanced interaction between p65 and ANXA2 (Fig. 4G). More importantly, the introduction of LINC01614^973–1775^ into the incubation system containing recombinant p65 and ANXA2 effectively enhanced their interaction (Fig. 4H).

To unravel the mechanisms involved in LINC01614-mediated glutamine influx, we performed GSEA for the mRNA microarray data of A549 cells treated with CAF exosomes. A panel of nuclear factor kappa B (NF-κB) target genes were significantly co-expressed with LINC01614 in CAF-exosome-treated A549 cells, including IL-6, CCL5, and CXCL10 (Fig. 4I), which was further verified by qPCR and ELISA both in CAF-exosome-treated A549 and ectopic LINC01614 expression A549 cells (Additional file 2: Fig. S5F-G). Similarly, upregulated NF-κB transcription activities and increased p65 nuclear translocation were observed in A549 cells treated with CAF exosomes, whereas knocking down LINC01614 in CAFs failed to active NF-κB transcription and p65 nuclear translocation (Fig. 4J–L). Moreover, ectopic expression of LINC01614 increased NF-κB transcription activities and p65 nuclear translocation in A549 cells (Fig. 4M–O). Together, these results indicated that CAF-derived LINC01614 enhanced p65 and ANXA2 interaction and promoted NF-κB activation.

### LINC01614 promotes ANXA2-dependent p65 phosphorylation and the transcription of SLC38A2 and SLC7A5

**Fig. 5:**
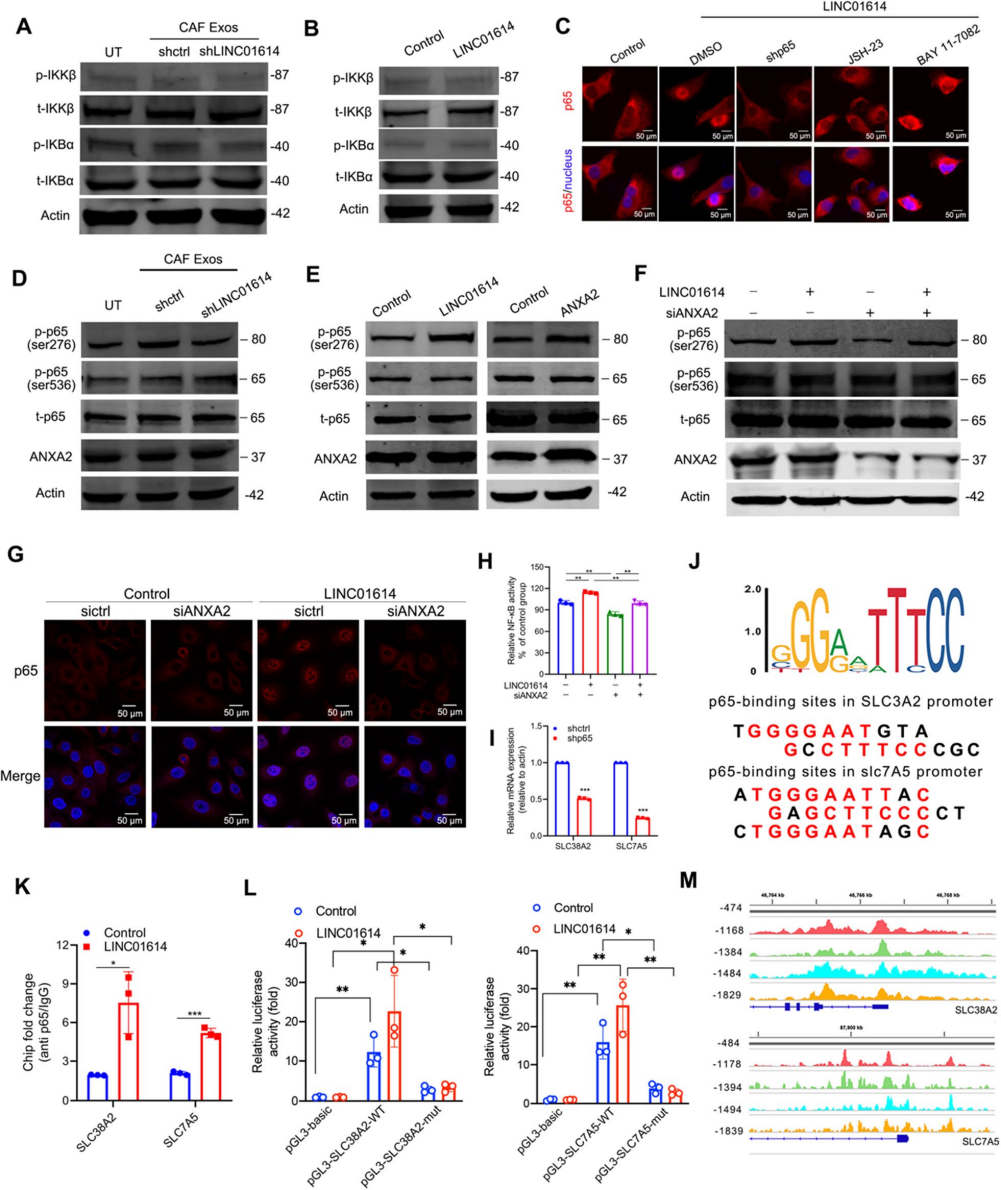
LINC01614 promotes ANXA2-dependent p65 phosphorylation and the transcription of SLC38A2 and SLC7A5. **A**, **D** Exosomes isolated from the CM of CAFs transduced with lenti-LINC01614-shRNA were added into A549 cells for 48 h. Exosomes from shctrl CAFs-treated A549 cells were used as controls. **A**, **B** Western blotting for total and phosphorylated IKK and IκBα in A549 cells with indicated treatments. **C** A549 cells were transduced without or with lenti-LINC01614 and pretreated with an inhibitor of NF-κB nuclear translocation (JSH-23) or IKK inhibitor (BAY 11-7-82). Immunofluorescent p65 staining showing its nuclear translocation in A549 cells (*n* = 3). **D** Expression of ANXA2, total, Ser276, and Ser536 phosphorylation of p65 in A549 cells with indicated treatments (*n* = 3). **E** Overexpressing LINC01614 or ANXA2 promoted Ser276 phosphorylation of p65 in A549 cells (*n* = 3). **F** Knockdown of ANXA2 abrogated the effects of LINC01614 on Ser276 phosphorylation (*n* = 3). **G** Representative immunofluorescent images of p65 nuclear translocation in A549 cells with indicated treatments. Scale bars, 50 μm. **H** NF-κB activity of A549 cells with indicated treatments. **I** qRT-PCR analysis of SLC38A2 and SLC7A5 in A549 cells with indicated treatments. **J** A conserved NF-κB binding element at the promoters of SLC38A2 and SLC7A5 were predicated by JASPAR (*n* = 3). **K** ChIP-PCR analysis for NF-κB occupancy at the promoters of SLC38A2 and SLC7A5 in A549 cells (*n* = 3). **L** Luciferase reporter assays of the transduced A549 cells transfected with reporter plasmids containing the SLC38A2 and SLC7A5 promoter, respectively. Wild type: -2000–0 construct; mutant: -2000–0 constructed with a point mutation at the NF-κB binding site. Transduced A549 cells transfected with a blank pGL3 plasmid used as a negative control (*n* = 3). **M** Graphic for ENCODE database of p65 ChIP-seq. For **H**, **I** and **K**, **L**, means ± s.d. are shown, and independent sample *t*-tests determined *P* values. *P < 0.05, **P < 0.01, ***P < 0.001. *UT* cancer cells without any treatment; *CM* conditioned medium; Exos, exosomes; *IP* immunoprecipitation; *LUAD* lung adenocarcinoma

To study the mechanisms by which LINC01614 activates the NF-κB pathway, we assessed the effect of LINC01614 on the phosphorylation of IκBα and IκB kinase (IKK). However, phosphorylation of IKK and IκBα was not influenced in A549 cells by ectopic LINC01614 expression or treated with CAF exosomes (Fig. 5A, B). Consistently, either inhibiting NF-κB nuclear translocation (JSH-23) or silencing p65, but not inhibiting IKK (BAY 11-7082), could abolish the effects of LINC01614 on p65 nuclear translocation (Fig. 5C). Moreover, silencing p65 also abolished the production of IL-6, CCL5, and CXCL10 (Additional file 2: Fig. S6A), suggesting that p65 nuclear retention was independent of upstream IKK or IκB activities. Because the phosphorylation of IKK and IκBα retained at low levels following LINC01614 treatment, we speculated that the p65 nuclear retention was independent of upstream IKK or IκBα activities. To further explore the mechanisms responsible for NF-κB activation in tumor cells, we detected the phosphorylation of p65, which was previously reported to maintain p65 nuclear retention [32]. Western blotting showed that CAF-derived exosomes enhanced p65 phosphorylation at the Ser276 residue, but not at the Ser536 residue in A549 cells. However, exosomes extracted from sh-LINC01614 transduced CAFs attenuated the enhancement of p65 phosphorylation at Ser276 (Fig. 5D). Moreover, LINC01614 or ANXA2 overexpression in A549 cells significantly enhanced p65 phosphorylation at Ser276 (Fig. 5E). ANXA2 was previously reported to mediate the phosphorylation-related activation of p65 NF-κB [33, 34]. Therefore, we determined whether LINC01614-induced p65 phosphorylation depended on ANXA2. As revealed by western blotting, immunofluorescent staining, and luciferase reporter assays, silencing ANXA2 attenuated LINC01614-induced p65 phosphorylation at Ser276, nuclear retention of p65, and NF-κB activation in A549 cells (Fig. 5F–H). Interestingly, silencing p65 reduced the expressions of SLC38A2 and SLC7A5 in A549 cells (Fig. 5I). We next assessed whether NF-κB activation promoted the transcription of SCL38A2 and SLC7A5. Sequence analysis by JASPAR suggested canonical binding motifs for NF-κB at the promoters of both SCL38A2 and SLC7A5 (Fig. 5J). Importantly, the NF-κB binding sites at the promoters of SLC38A2 and SLC7A5 were confirmed, respectively, by immunoprecipitation (ChIP) analysis with the anti-p65 antibody and the luciferase reporter assay (Fig. 5K, L). Moreover, encoded ChIP-sequencing data confirmed the peaks of p65 at the promoters of SLC38A2 and SLC7A5 in the genomic positions as revealed by our ChIP-PCR analysis (Fig. 5M).

Furthermore, inhibiting NF-κB nuclear translocation (JSH-23), but not inhibiting IKK (BAY 11-7082), could abolish the effects of LINC01614 on the expression of SLC38A2 and SLC7A5 (Additional file 2: Fig. S6B). Silencing ANXA2 in A549 cells attenuated LINC01614-induced SLC38A2 and SLC7A5 upregulation (Additional file 2: Fig. S6C-D) and partially reversed the enhanced oxidative phosphorylation and glutamine influx capacity of ectopic LINC01614 expression in A549 cells (Additional file 2: Fig. S6E-F). These data suggest that CAF-derived LINC01614 interacts with p65 and ANXA2 and promotes ANXA2-induced phosphorylation of p65 at Ser276, leading to NF-κB activation of the transcription of two glutamine transporters, SLC38A2 and SLC7A5, in LUAD cells.

### Proinflammatory cytokines released from tumor cells upregulate LINC01614 in CAFs in a feedforward loop

We performed serial experiments to further investigate the upstream mechanisms by which LINC01614 is upregulated in CAFs. We determined the expression of LINC01614 in CAFs of different cell generations and found that the expression of LINC01614 in CAFs gradually decreased with culture generations (Additional file 2: Fig. S7A). However, the expression of LINC01614 in CAFs increased with time when co-cultured with A549 cells. Interestingly, A549 cells failed to upregulate LINC01614 expression in CAFs when silencing LINC01614 or inhibiting CAF exosome secretion (Additional file 2: Fig. S7B), suggesting the transmission of LINC01614 from CAFs to cancer cells may form regulatory positive feedforward loops for LINC01614 upregulation in CAFs. Because the production of NF-κB target genes IL-6, CCL5, and CXCL10 in A549 cells were upregulated by CAF-derived LINC01614, we evaluated whether IL-6, CCL5, or CXCL10 increased LINC01614 expression. We observed that treating A549 CM with neutralizing antibodies against IL-6 and CXCL10, but not against CCL5, strongly abrogated LINC01614 upregulation in CAFs (Additional file 2: Fig. S7C). Moreover, CM from LINC01614 overexpressed A549 cells upregulated the expression of LINC01614 in CAFs (Additional file 2: Fig. S7C). Furthermore, recombinant IL-6 and CXCL10 were sufficient to induce LINC01614 overexpression in CAFs (Additional file 2: Fig. S7D). These results suggest that IL-6 and CXCL10 produced by LUAD cells induce LINC01614 upregulation in CAFs. Given the specific cellular origin for LINC01614, we hypothesized that IL-6 and CXCL10 induced fibroblast lineage-specific factors that could be responsible for LINC01614 transcription in CAFs, which warrants further study. To investigate the potential competition relationship between CAFs and LUAD cells, we assessed glutamate and glutaminase content in CAFs. As shown in Additional file 2: Fig. S4L and Additional file 2: Fig. S7E, neither silencing/overexpressing LINC01614 nor IL-6 treatment had no effect on the glutamine uptake of CAFs. These results suggest that LINC01614 upregulating in CAFs did not result in increased glutamine uptake and potential ‘competition’ with LUAD cells.

### CAF-derived LINC01614 promotes LUAD tumorigenesis in vivo and is a potential therapeutic target

**Fig. 6:**
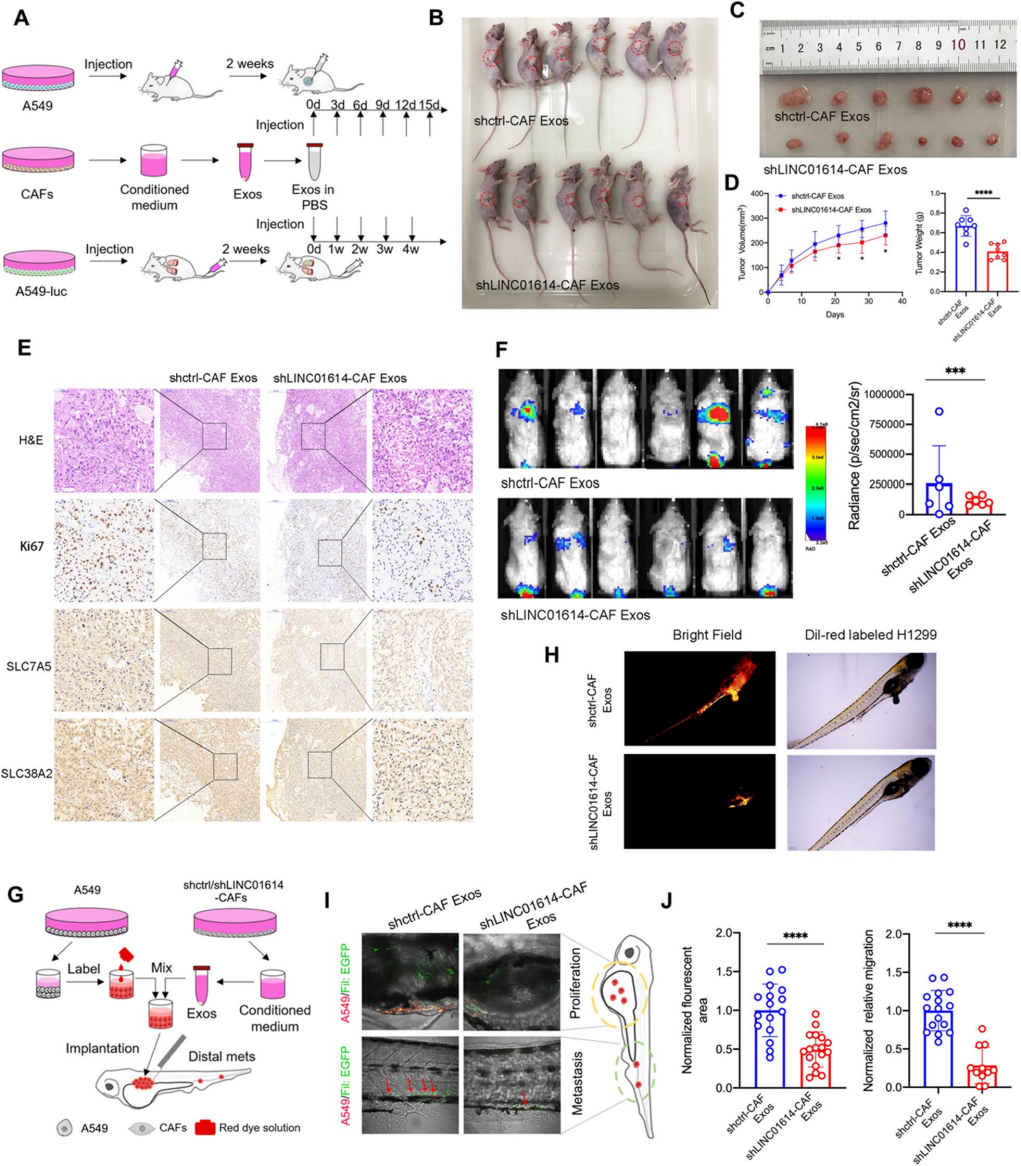
CAFs release LINC01614 in exosomes to enhance glutamine uptake and progression of LUAD in vivo. **A** Schematic diagram of xenograft and subcutaneous tumorigenicity in mice. **B–E**, Nude mice were subcutaneously xenografted with A549 cells (1 × 10^7^ cells) and treated intratumorally with CAF-derived exosomes (0.5 μg kg^−1^) every 3 d for 2 weeks (*n* = 6 mice per group). **B** Images of tumor engraftment in nude mice. **C**, **D** Tumor weight and growth curves. **E** Representative H&E staining and IHC staining for Ki67, SLC38A2, and SLC7A5. **F** NCG (NOD prkdc^−/−^IL-2Rg^−/−^) mice were xenografted with A549-luc cells (5 × 10^6^ cells) through tail vein injection and treated intravenously with CAF-derived exosomes once a week for 4 weeks (*n* = 6 mice per group). Representative bioluminescent images for lung metastasis. Scanning was performed 8 weeks after tumor implantation. **G** Schematic diagram of implantation of cancer cells in zebrafish embryos (Fli1:EGFR). A549 cells were labeled with Dil implanted into the perivitelline space of each zebrafish. After 24 h, the exosomes (5 ng) isolated from CAFs transduced without (shctrl) or with shRNA for LINC01614 (shLINC01614) were injected into the (Fli1:EGFR) zebrafish embryos. Dissemination of A549 was monitored. **H** Representative images of Dil-red-labeled A549 cells in zebrafish embryos with indicated treatments. **I** Representative confocal microscopy images of the dissemination of implanted DiD-labeled A549 cells in zebrafish embryos (Fli1:EGFP) injected with CAF-derived exosomes (5 ng) (*n* = 15). The approach schema is illustrated. **J** Quantification of total numbers of disseminated and metastatic cells in the primary tumor surroundings (upper) and the trunk regions (lower) of zebrafish. For **D**, **J** means ± s.d. are shown, and independent sample *t*-tests determined *P* values. *P < 0.05, **P < 0.01, ***P < 0.001. *Exos* exosomes, *LUAD* lung adenocarcinoma

To investigate the tumor-promoting roles of exosome-packaged LINC01614 in vivo, we inoculated A549 cells with or without a co-injection of CAFs into the single flank of nude mice. The xenograft tumor models showed that an intratumor injection of CAF exosomes or co-injection of CAFs promoted lung cancer growth (Fig. 6A–D, Additional file 2: Fig. S8A-D). IHC staining revealed that xenografts from the mice with the exosome injection exhibited more Ki67 and SLC38A2- and SLC7A5- positive cells. Conversely, exosomes from sh-LINC01614 transduced CAFs inhibited tumor growth in the xenografts, accompanied by a reduced expression of Ki67, SLC38A2, and SLC7A5 (Fig. 6E). Moreover, CAF exosome treatment promoted lung metastasis of LUAD cells in NCG (NODprkdc^−/−^ IL-2Rg^−/−^) mice, which was partially reversed by silencing LINC01614 in CAFs (Fig. 6F). In addition, we used zebrafish tumor models to study tumor growth and metastasis, particularly to decipher the initial steps of the metastatic cascade regulated by exosome-packaged LINC01614. Co-implantation of LUAD cells with CAFs or CAF-derived exosomes sharply enhanced the number of proliferative and metastatic cancer cells. Interestingly, CAFs also exhibited “metastatic” capacity in the zebrafish body (Fig. 6G–J; Additional file 2: Fig. S8E-F). However, silencing LINC01614 in CAFs before co-injection or exosome extraction attenuated the number of metastatic cancer cells and CAFs (Fig. 6G–J; Additional file 2: Fig. S8E-F).

### LINC01614 correlates with glutamine transporters and poor survival of patients with LUAD

**Fig. 7:**
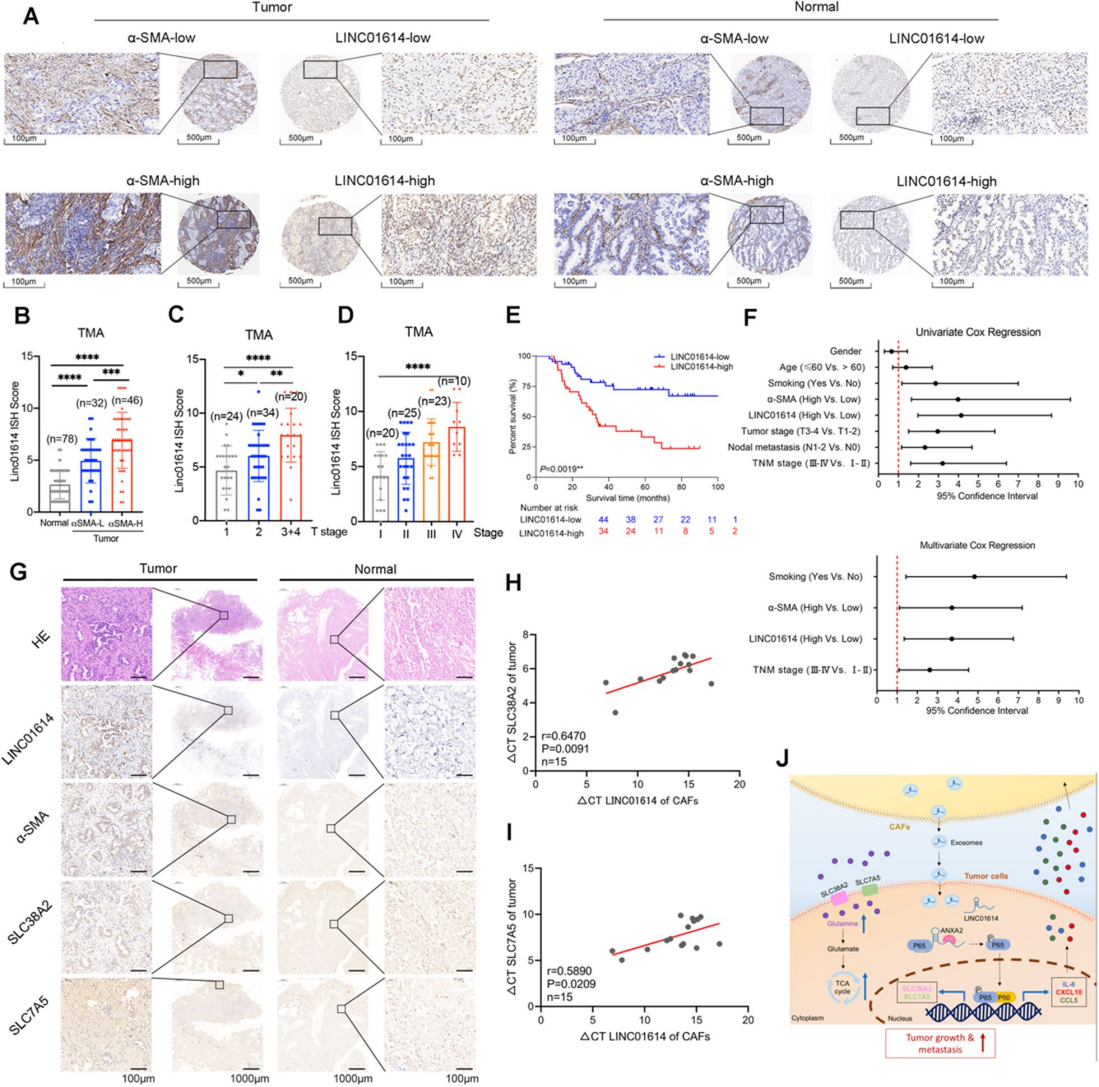
LINC01614 correlates with glutamine transporters and poor survival in patients with LUAD. **A** Representative ISH for LINC01614 and IHC for α-SMA in a TMA cohort containing 78 paired LUAD tissues and adjacent normal tissues. Scale bars, 500 μm (×100 magnification), 100 μm (×400 magnification) (*n* = 78). **B–D** The CISH results of the TMA. **B** The expression of LINC01614 was upregulated in the α-SMA High expression (high CAFs infiltration) group (≥ 6, median). **C**, **D** The expression level of LINC01614 in LUAD tissues was positively correlated with the T stage and TNM stage in the TMA cohort. **E** Higher expression (≥ 6, median) of LINC01614 in LUAD tissues was associated with poor prognosis in the TMA cohort. **F** Univariate and multivariate Cox regression analyses indicated that high expression of LINC01614 in LUAD tissues was an independent prognostic factor for poor survival (*n* = 78). **G** Representative H&E staining, ISH for LINC01614, and IHC for α-SMA, SLC38A2, and SLC7A5 in LUAD and adjacent normal tissues (*n* = 10). **H** Correlation between LINC01614 expression in CAFs and SLC38A2 in tumor cells from the same patients (*n* = 15). **I** Correlation between LINC01614 expression in CAFs and SLC7A5 in tumor cells from the same patients (*n* = 15). **J** Graphical illustration of the interaction between CAFs and LUAD cells. For **B–D**, the mean ± s.d. are shown, and Student’s *t*-test determined *P* values. For **H**, **I** Spearman correlation analysis was performed. **P < 0.01, ***P < 0.001, ****P < 0.001. LUAD, lung adenocarcinoma

To determine whether our findings were clinically relevant, we analyzed the correlation of LINC01614 expression with CAF infiltration, as revealed by immunostaining for α-SMA in a TMA containing 78 pairs of LUAD and adjacent normal tissues. We observed that LINC01614 was abundantly expressed in CAFs. More importantly, 37 of 78 (47.4%) LUAD patients with abundant CAF infiltration exhibited enhanced hybridization signals of LINC01614 in the tumor cells (Fig. 7A). Moreover, CISH scores of LINC01614 in tumors were positively associated with the density of CAFs (Fig. 7B). High LINC01614 expression was correlated with tumor size, histological grading, and staging (Fig. 7C, D). Kaplan–Meier survival analysis revealed that high expression of LINC01614 was associated with poor overall survival of patients with LUAD (Fig. 7E). The Cox proportional hazards model indicated that LINC01614 was an independent prognostic factor for LUAD (Fig. 7F).

Furthermore, we correlated LINC01614 expression with tumor glutamine transporters, as detected by immunostaining for SLC38A2 and SLC7A5 and CAF infiltration as denoted by α-SMA immunostaining in 10 cases of patients with LUAD. Consistently, LINC01614 was abundant in CAFs, and LINC01614 expression of tumor cells was positively correlated with the density of α-SMA^+^ CAFs and the expression of SLC38A2 and SLC7A5 in tumors (Fig. 7G). In addition, the levels of LINC01614 in primary CAFs positively associated with the mRNA levels of SLC38A2 and SLC7A5 in the paired LUAD tumor cells (Fig. 7H, I). Altogether, these clinical data are consistent with the experimental finding that LINC01614 is transmitted from CAFs to LUAD cells, thereby enhancing the glutamine uptake in LUAD.

Next, we determined the correlation of LINC01614 expression with the clinicopathological status of patients with LUAD from TCGA dataset and Jiangsu Cancer Hospital (JSCH) cohort. LINC01614 was significantly upregulated in LUAD tissues in TCGA dataset and JSCH cohort. High LINC01614 expression in LUAD tissues was correlated with histological staging and lymph node metastasis. Furthermore, high expression of LINC01614 was associated with poor prognosis of patients with LUAD in TCGA cohort (Additional file 2: Fig. S9A-G). These results suggest that LINC01614 could be a potential biomarker and therapeutic target for LUAD.

## Discussion

Metabolic reprogramming allows cancer cells to maintain proliferation and overcome metabolic challenges related to oxygen and nutrient limitations [35]. Metabolic reprogramming of cancer cells has long been studied in regard to how cancer cells utilize glucose via aerobic glycolysis. Recently, cancer cells have been shown to utilize glutamine and other nutrient sources for survival [3], with some cancer cells even preferring the “addicting” glutamine over glucose [4]. However, how and why cancer cells preferentially utilize specific addicting nutrients remains unknown. In this study, we identified a CAF-specific lncRNA-LINC01614 that facilitates cancer cell addiction to glutamine in LUAD. Mechanistically, LINC01614 can be released and transmitted by exosomes to LUAD cells, enhancing glutamine uptake and the progression of LUAD cells. LINC01614 can induce NF-κB activation via p65 phosphorylation of LUAD cells, promoting the secretion of IL-6 from LUAD to upregulate LINC01614 in CAFs, constituting a feedforward loop between CAFs and LUAD cells.

CAFs are one of the most abundant stromal components in the TME. An increasing number of studies have shown that CAFs play important roles in cancer pathogenesis, which has prominent clinical implications [6, 36]. Recent studies demonstrated that CAFs participate in TME metabolic remodeling through exosomes [37] and amino acid secretion [38, 39]. Here, we found that CAFs produce exosomes to transmit lncRNA and facilitate LUAD cells addiction to glutamine. More importantly, LINC01614 was selectively expressed in CAFs. LncRNAs are notably shown to exhibit cell lineage- and developmental stage-dependent expression patterns. Several cell lineage-specific lncRNAs were identified [30], and some controlled lineage-specific regulatory programs and regulated pathophysiological processes [11, 30, 40, 41]. Two of the latest studies demonstrated that IRENA and HISLA are specifically expressed in tumor-associated macrophages (TAMs) in breast cancer [11, 41]. However, LINC01614 has been reported as an oncogene and biomarker in several cancers, including gastric cancer [42], esophageal squamous cell carcinoma [43], breast cancer [44], and LUAD [45]. The specificity and spatial cellular expression patterns of LINC01614 were not addressed in these studies. Liu et al. mentioned that LINC01614 was upregulated in LUAD cells, whereas their results showed that LINC01614 was only upregulated by 1.7-fold in LUAD cells (H1395 and H1975) compared with human normal bronchial epithelium cells (BEAS-2B) [45]. However, our data showed that LINC01614 was significantly upregulated in CAFs (up to tenfold) but not in LUAD cell lines or other primary TME cells. We further confirmed the spatial cellular expression of LINC01614 by FISH staining and flow cytometry. Our data extended the understanding of this emerging field by demonstrating a CAF-specific lncRNA LINC01614 could be encapsulated in exosomes and shuttled from CAFs to drive the preferential uptake of glutamine over glucose in LUAD cells.

Exosomes contain abundant noncoding RNAs, including microRNAs, lncRNAs, and ribosomal RNAs [28]. Several studies suggested that exosomal lncRNAs were involved in chemoresistance [19], metastasis [46], and glycolysis [11] in various cancers. We demonstrated that LINC01614 is released via exosomes from CAFs to LUAD cells. Mechanistically, LINC01614 directly interacts with ANXA2 and p65 in LUAD cells, for which LINC01614 serves as a scaffold to facilitate the phosphorylation and activation of NF-κB. Notably, NF-κB activation is not a result of IKK and IκB phosphorylation. Rather it relies on the post-translational modification of p65. ANXA2 was reported to promote p65 phosphorylation and NF-κB activation [33]. Here, we found that LINC01614 enhanced the interaction between p65 and ANXA2, and the LINC01614-induced p65 phosphorylation was ANXA2 dependent. We speculated that ANXA2 could lead to a conformational change in p65, enhancing its phosphorylation. Our study showed that exosomal lncRNA could mediate metabolic reprogramming between stromal and cancer cells. Moreover, primary tumor-released exosomes could circulate to distant organs to establish a premetastatic niche [47, 48]. In this scenario, CAF-produced exosomes may fuel metastasis and colonization of cancer cells to a distal metastatic niche, which warrants additional studies.

Metabolic reprogramming of cancer cells could be exploited as a therapeutic target [49]. Our study demonstrated that targeting CAF-specific LINC01614 inhibits glutamine uptake and the progression of LUAD cells, highlighting that CAF-specific lncRNAs could serve as an attractive target in cancer treatment. Although small-molecule inhibitors that suppress glutamine transporters and glutaminase have exhibited synergistic effects with multiple anti-cancer agents in murine models [50–52], because of the intrinsic metabolic heterogeneity and flexibility within the TME, these efforts have met with limited success in the clinical setting [52–54]. Our data suggest that molecular therapy targeting LINC01614 could provide a more specific and feasible approach to inhibit glutamine metabolism in LUAD cells. Recent studies have shown that peptide-, lipid-, and nanoparticle-based siRNA delivery systems could be exploited to improve tumor targeting and safety [43, 44], providing promising opportunities to explore lncRNAs as therapeutic targets. The strategy of choosing a precise delivery system to mediate specific LINC01614 knockdown in CAFs for cancer treatment warrants further study.

In summary, this study revealed that CAFs preferentially promote the addiction of cancer cells to glutamine via upregulation of amino acid transporters in LUAD. The CAF-specific lncRNA LINC01614 determines the effect of metabolic reprogramming and is an example of how an exosome-transmitted lncRNA promotes the activation of NF-κB signaling and forms a feedforward loop between CAFs and LUAD cells. Notably, our study has highlighted the therapeutic potential of targeting a CAF-specific lncRNA to inhibit glutamine utilization and reverse tumor-promoting activities of LUAD.

## Supplementary Information

**Additional file 1.** Supplementary Tables.

**Additional file 2.** Supplementary Figures.

## Data Availability

Source data for figures and Additional file 2 have been provided as Statistics Source Data. The RNA-seq data have been submitted to the Gene Expression Omnibus (https://www.ncbi.nlm.nih.gov/geo/), and the data can be accessed by the accession number GSE213043. Other data supporting the findings of this study are available from the corresponding authors.
