# Supplementary material for: Cancer-associated fibroblast-specific lncRNA LINC01614 enhances glutamine uptake in lung adenocarcinoma
Source: J Hematol Oncol. 2022 Oct 8;15:141. doi: 10.1186/s13045-022-01359-4 (PMC9548164; doi:10.1186/s13045-022-01359-4)
Supplement: Supplementary file 2 — Additional file 2. Supplementary Figures. [file 13045_2022_1359_MOESM2_ESM.docx]

**
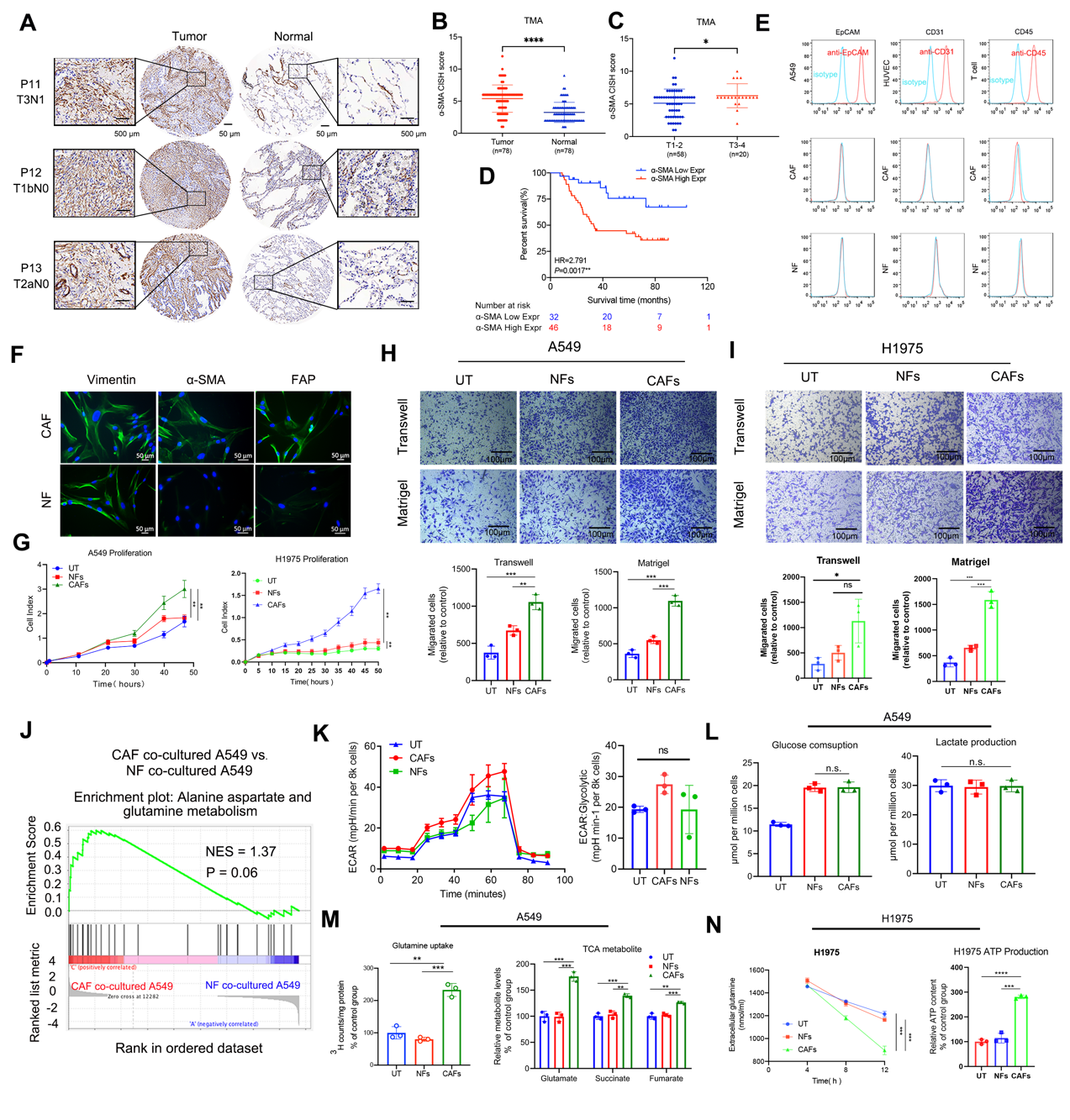
**

**Fig. S1 CAFs promote LUAD progression and enhance glutamine metabolism of LUAD cells. A,** Representative images of IHC staining for α-SMA in a TMA cohort containing 78 paired LUAD tissues and adjacent normal tissues. Scale bars, 500 μm (× 100 magnification), 50 μm (× 400 magnification). **B**, CISH results of the TMA. **C**, Expression level of α-SMA was positively correlated with the T stage in the TMA cohort. **D**, Higher expression (≥ 6, median) of α-SMA is associated with poor prognosis in the TMA cohort. **E**, CAFs and NFs isolated from patients with LUAD were negative for EpCAM (epithelial marker), CD31 (endothelial marker), and CD45 (leukocyte marker), assessed by flow cytometry. A549 LUAD cells, human umbilical vein cells (HUVECs), and human T lymphocytes were used as positive controls. Representative images are shown. **F**, Representative images show that CAFs and NFs isolated from LUAD cells expressed a high level of myofibroblast markers, α-SMA and FAP. Scale bars, 50 μm. **G-I**, LUAD cells (A549 and H1975) were co-cultured with NFs or CAFs in Transwell systems. **G**, RTCA was used to detect the proliferation of LUAD cells (n= 3). **H-I**, Transwell and Matrigel assays show that CAFs significantly increased the migration and invasion abilities of A549 (H) and H1975 cells (I) (n= 3). **J**, KEGG-based GSEA revealed an enrichment of “Alanine aspartate and glutamine metabolism” genes in the A549 cells co-cultured with CAFs compared to NFs co-cultured A549 cells (n= 3). **K**, ECARs of A549 cells with indicated treatments (n= 3). **L,** Glucose consumption and lactate production in A549 cells with indicated treatments (n= 3). **M-N**, Glutamine uptake, TCA metabolites, extracellular glutamine levels, and ATP production of LUAD cells with indicated treatments (n= 3). For **G-I** and **K-N**, means ± s.d. are shown, and independent sample *t*-tests determined *P* values. * *P* < 0.05, ** *P* < 0.01, *** *P* < 0.001. UT, cancer cells without treatment; CM, conditioned medium; Exos, exosomes; RTCA, Real-time xCELLigence analysis; LUAD, lung adenocarcinoma.


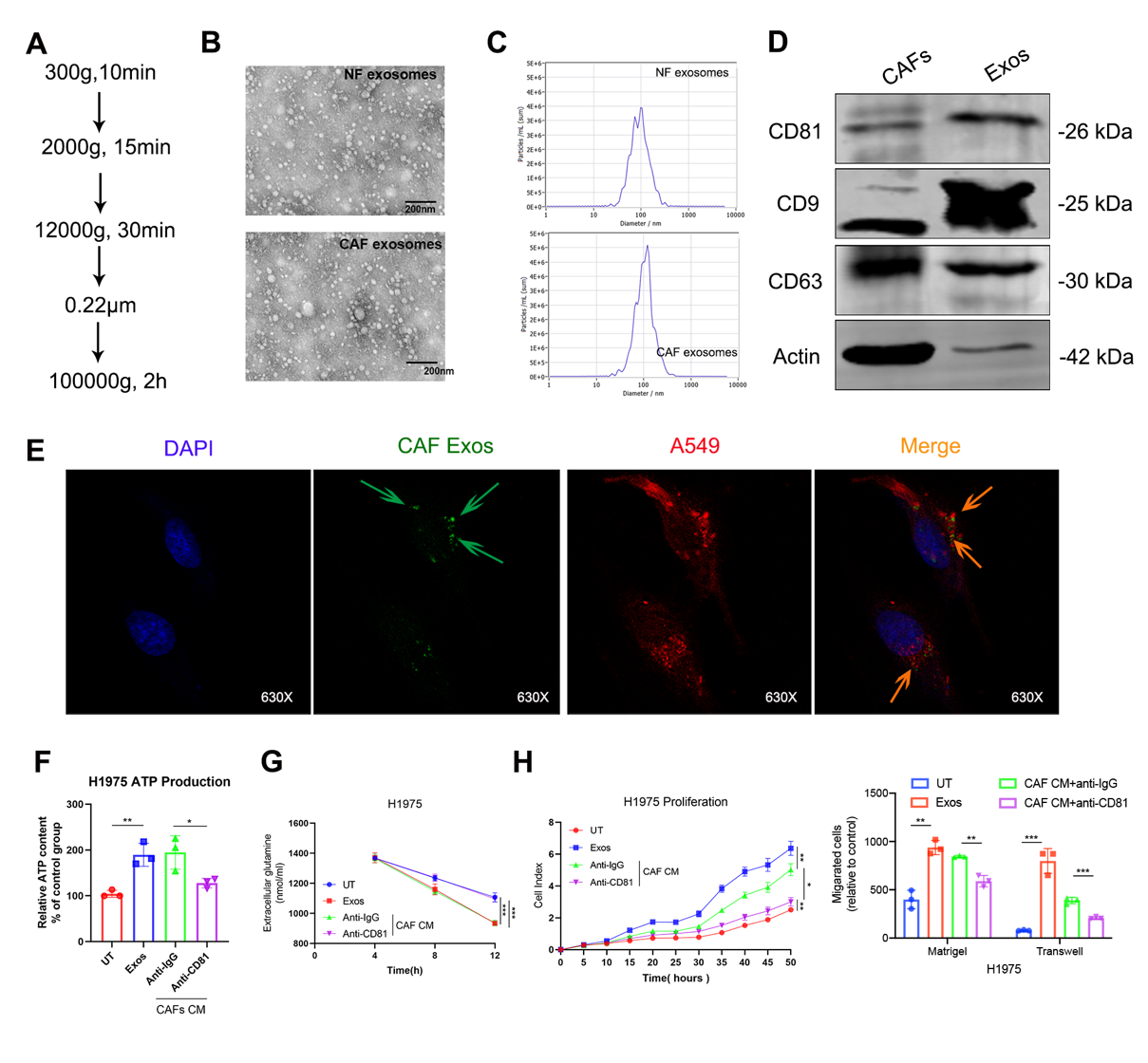


**Fig. S2 Exosome-packaged RNA from CAFs enhances the glutamine uptake and progression of LUAD cells. A,** Schematic of the procedure for exosome purification. **B**, Representative electron microscopy image of exosomes extracted from NF and CAF CM. Scale bar, 100 nm. **C**, Particle diameters of exosomes isolated from NF and CAF CM were determined by NanoSight. **D**, Western blotting for the expression of CD81, CD9, and CD63 in the CAFs or exosomes isolated from the CAF CM (n = 3). **E**, Fluorescence microscopy image showing the delivery of Dio-labeled exosomes (green) to Dil-labeled A549 cells (red). Orange arrows indicate representative delivered exosomes (n= 3)). **F**, ATP production (n = 3) of H1975 cells with indicated treatments. **G**, Extracellular glutamine levels in H1975 cells cultured for 4, 8, and 12 h with indicated treatments. **H**, RTCA proliferation assays, Transwell and Matrigel assays of H1975 cells with indicated treatments. For **F-H**, Mean ± s.d., n.s, nonsignificant by Student’s *t*-test. ** *P* < 0.01, *** *P* < 0.001, n.s., nonsignificant. UT, cancer cells without treatment; CM, conditioned medium; Exos, exosomes; RTCA, Real-time xCELLigence analysis; LUAD, lung adenocarcinoma.

**
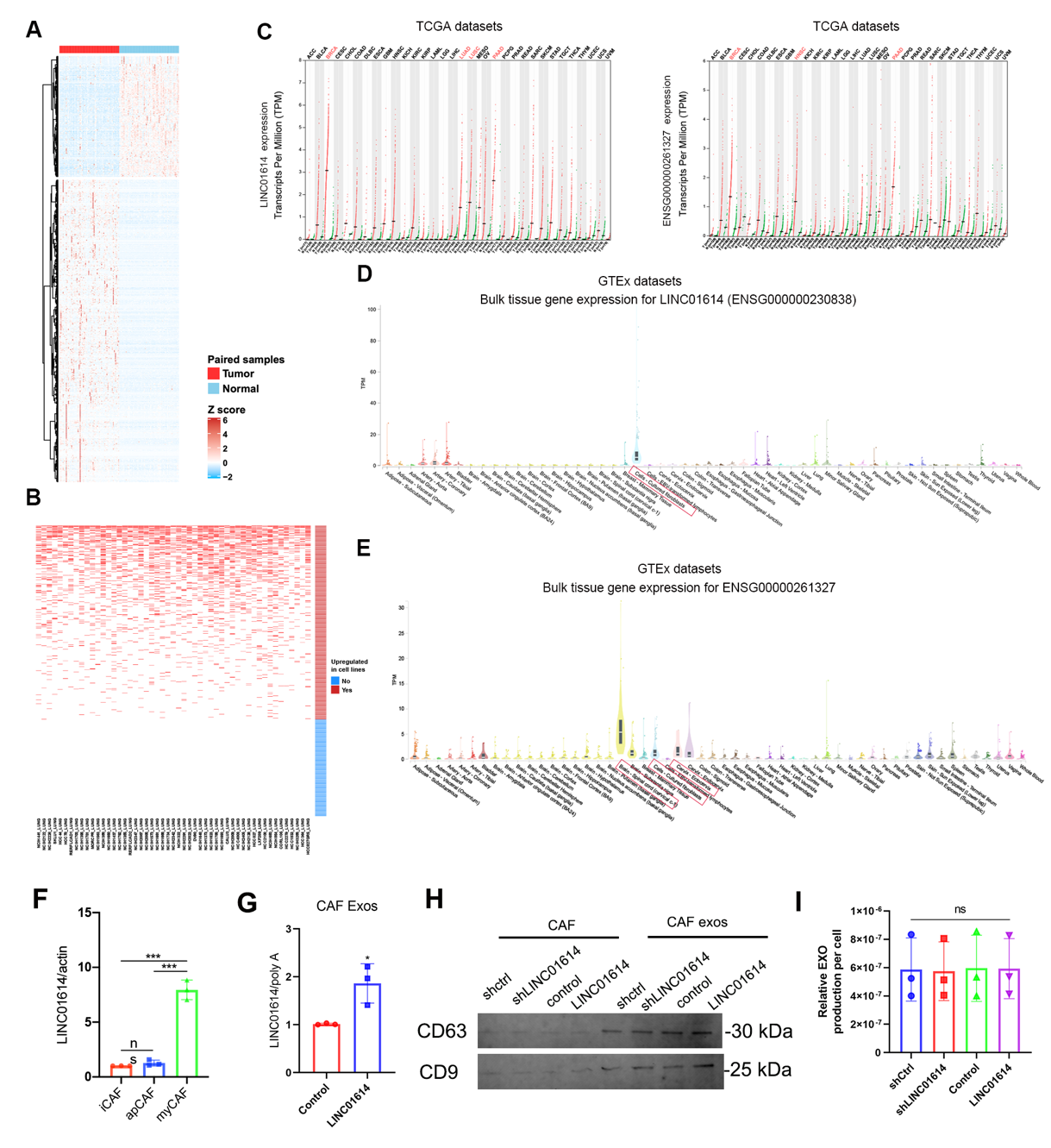
**

**Fig. S3 Identification of CAF-specific lncRNAs. A,** Differentially expressed genes between LUAD tissues and normal lung tissues from TCGA dataset. **B**, A total of 195 lncRNAs highly expressed in LUAD tissues from TCGA dataset but not in any LUAD cell lines based on the CCLE database. **C**, LINC01614 and ENSG00000261327 expression in TCGA dataset. **D-E**, LINC01614 and ENSG00000261327 expression in the GTEx dataset. **F**, qRT-PCR analysis of LINC01614 in different CAFs subsets. **G**, qRT-PCR analysis of LINC01614 in exosomes derived with indicated CAFs. **H**, Western blot for the expression of CD9, and CD63 in the CAFs or exosomes isolated from the CAF CM. **I**, Relative exosome production per cell of CAFs with indicated treatments. For **F-I**, Mean ± s.d., n.s, nonsignificant by Student’s *t*-test. ** *P* < 0.01, *** *P* < 0.001, n.s., nonsignificant .myCAF, myofibroblasts; iCAF, inflammatory CAF; apCAF, antigen-presenting CAF; CCLE, Cancer Cell Line Encyclopedia; GTEx, Genotype-Tissue Expression. LUAD, lung adenocarcinoma.

**
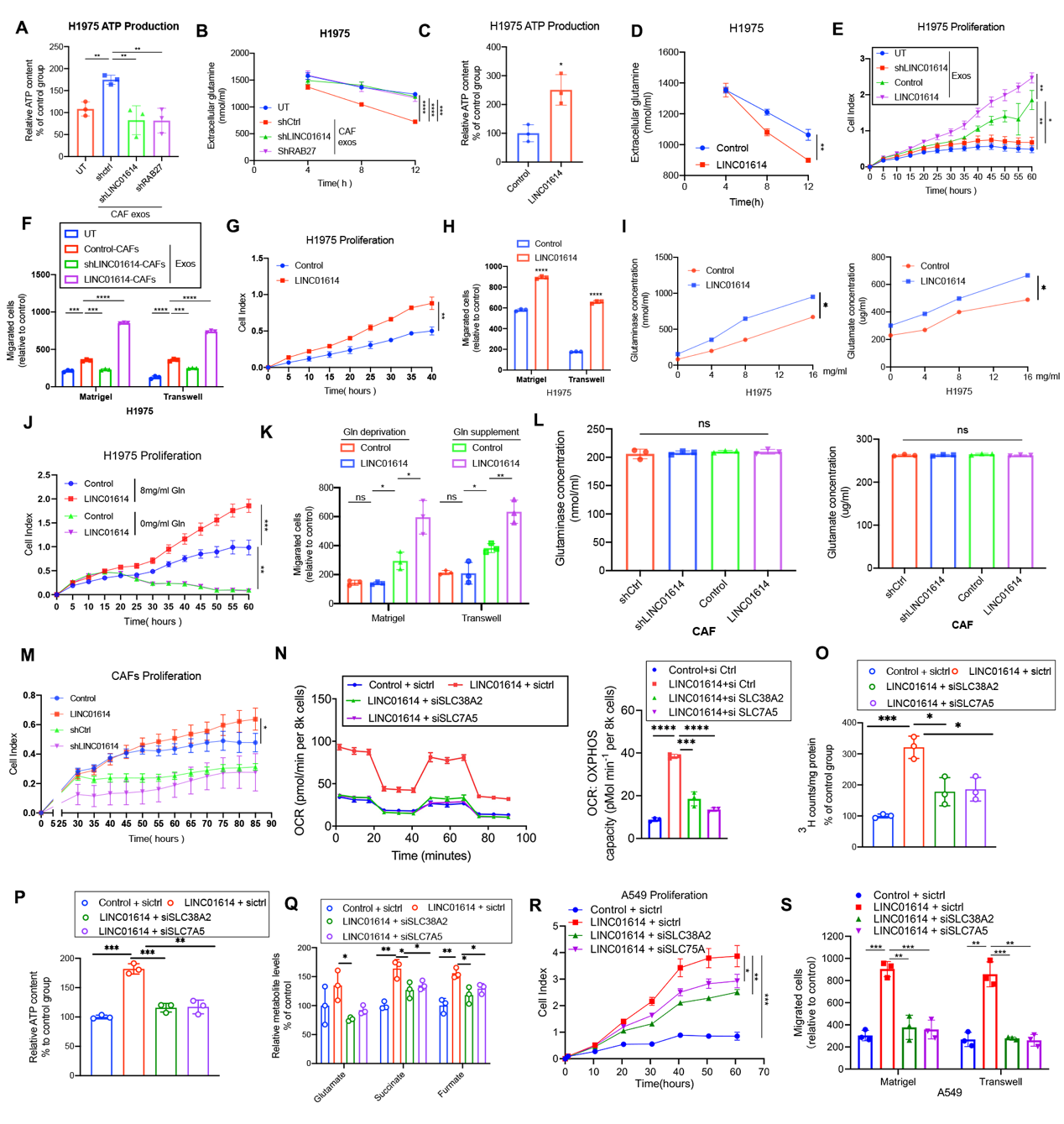
**

**Fig. S4 CAF exosome-packaged LINC01614 enhances glutamine influx and the progression of LUAD cells via upregulation of SLC38A2 and SLC7A5. A-D**, ATP production and extracellular glutamine levels of H1975 cells with indicated treatments. **E-H**, RTCA proliferation assays, Transwell and Matrigel assays of H1975 cells with indicated treatments. **I**, Glutaminase and glutamate content of H1975 cells with indicated treatments. **J-K**, RTCA proliferation, Migration and invasion assays of H1975 cells with indicated treatments. **L-M**, Glutaminase and glutamate content, RTCA proliferation of CAFs with indicated treatments. **N-S,** Lenti-LINC01614 transduced A549 cells were transfected with si SLC38A2 or si SLC7A5 and then used for the indicated experiments. **N**, OCR of the A549 cells with indicated treatments (n = 3). **O-P**, ^3^H-glutamine uptake and ATP production of A549 cells with indicated treatments (n= 3). **Q,** Glutamine-derived TCA cycle intermediates in A549 cells with indicated treatments (n= 3). **R**, Proliferation ability of A549 cells was detected by RTCA (n = 3). **S,** Transwell and Matrigel assays of the A549 cells transfected with SLC38A2 and SLC7A5 siRNAs. For all experiments, the mean ± s.d. are shown, and Student’s *t*-test determined *P* values. ** *P* < 0.01, *** *P* < 0.001, n.s., nonsignificant. UT, cancer cells without any treatment; CM, conditioned medium; Exos, exosomes; LUAD, lung adenocarcinoma; RTCA, Real-time xCELLigence analysis; LUAD, lung adenocarcinoma.

**
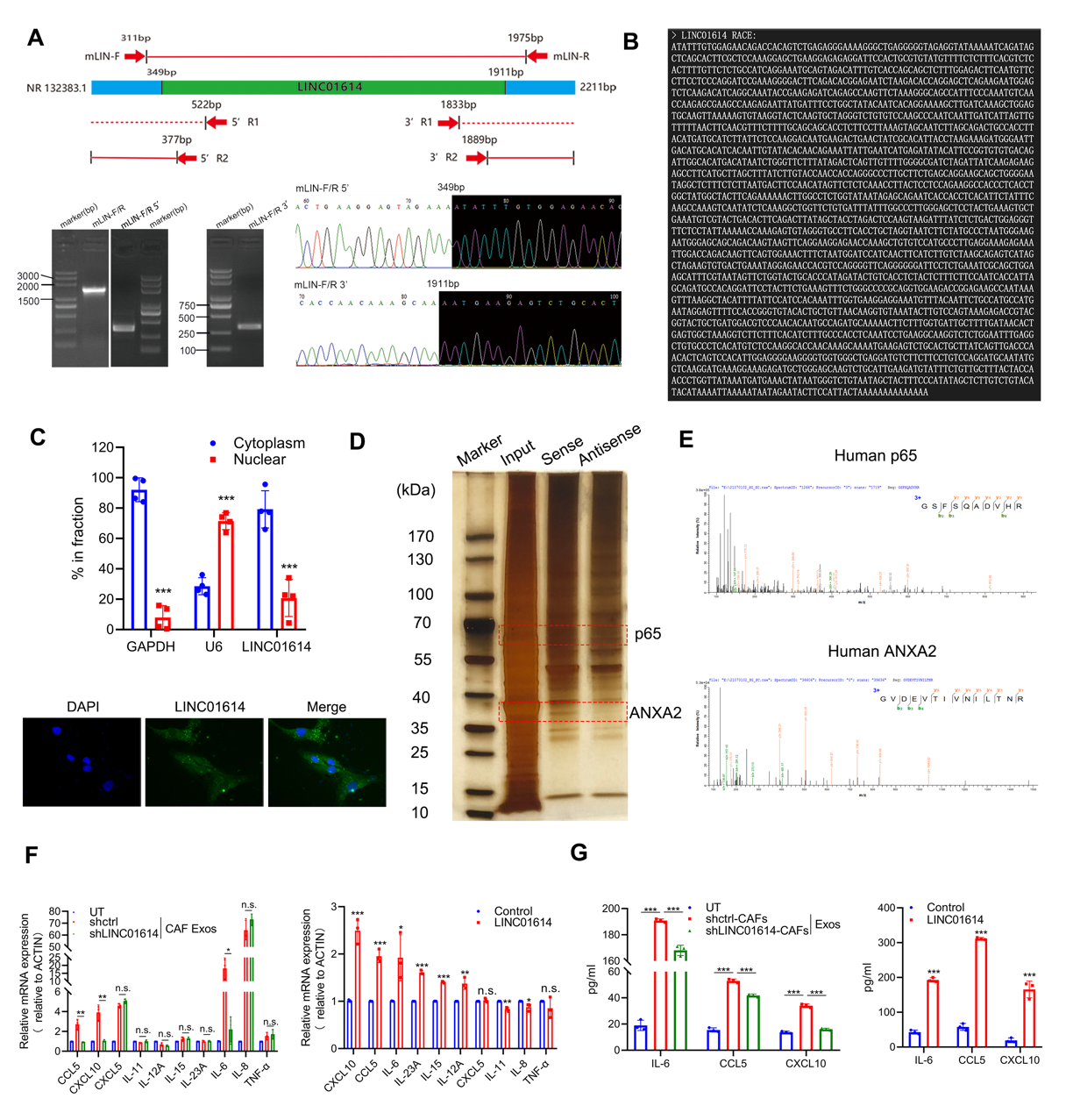
**

**Fig. S5. LINC01614 associates with ANXA2 and p65 to promote NF-κB activation. A-B**, Agarose gel and Sanger sequencing assays showed the full-length of LINC01614 and the nested PCR product of LINC01614 RACE analysis. **C**, Nuclear mass separation and fluorescence *in situ* hybridization (FISH) assays indicate that LINC01614 is distributed primarily in the cytoplasm of CAFs (n = 3). **D**, RNA pull-down assay followed by silver staining revealed proteins associated with biotinylated LINC01614 or antisense in A549 cells. **E,** Mass spectrometry analysis for the peptides of LINC01614-interaction with p65 and ANXA2. **F**, qRT-PCR verified that a panel of NF-κB target genes was upregulated in CAF exosomes treated A549 cells and lenti-LINC01614 transduced A549 cells, including IL-6, CXCL10, and CCL5 (n = 3). **G**, IL-6, CXCL10, and CCL5 levels in A549 CM with indicated treatments were determined by ELISA. For **F-G**, Mean ± s.d. are shown, and Student’s *t*-test determined *P* values. * *P* < 0.05, *** *P* < 0.001 by Student’s *t*-test. UT, cancer cells without any treatment; CM, conditioned medium; Exos, exosomes; RTCA, Real-time xCELLigence analysis; LUAD, lung adenocarcinoma; LUAD, lung adenocarcinoma.


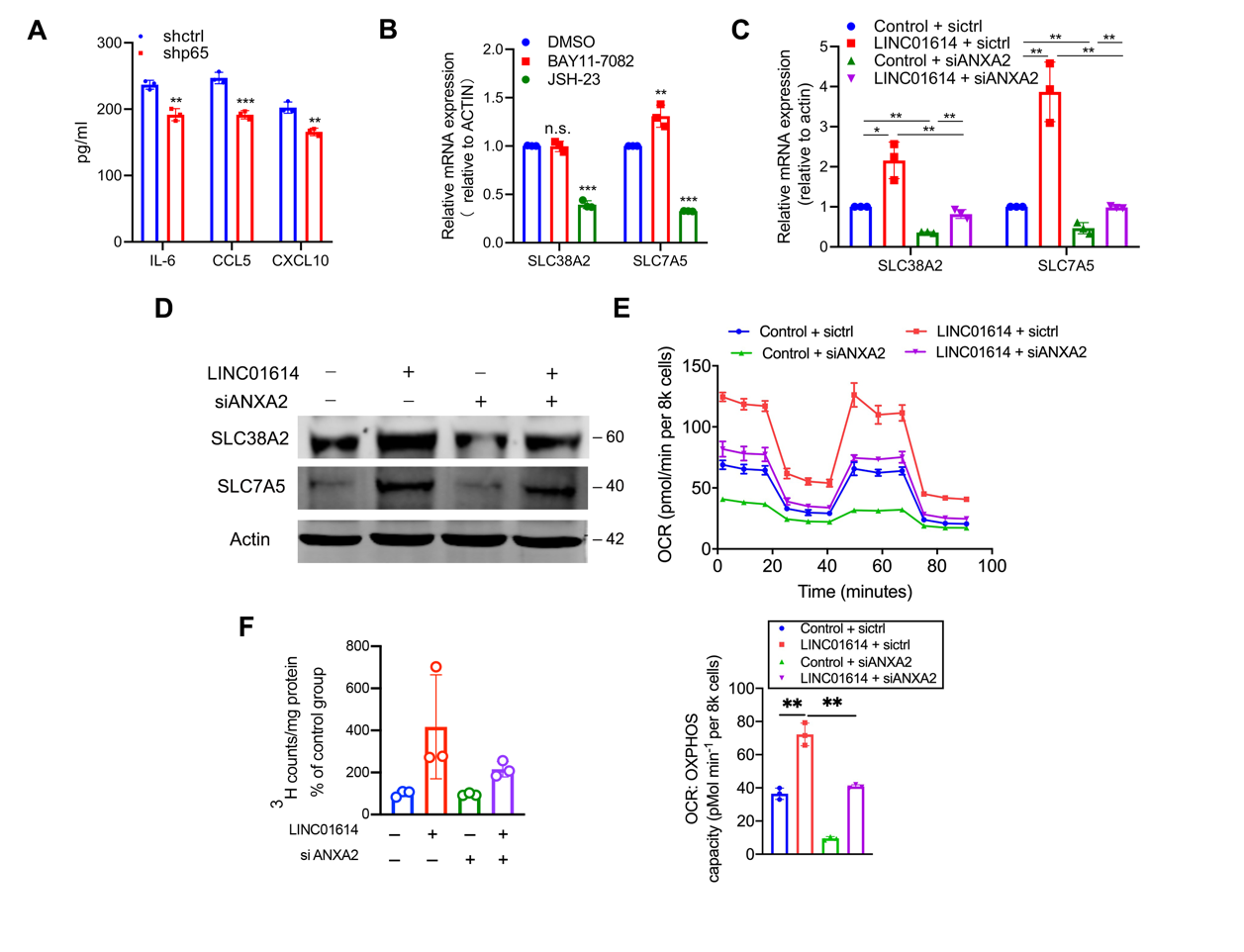


**Fig. S6 LINC01614 binds to and phosphorylates p65 in an ANXA2 dependent way. A**, ELISA analysis of IL-6, CXCL10, and CCL5 in LINC01614-transduced A549 cells with indicated treatments. **B**, qRT-PCR analysis of SLC38A2 and SLC7A5 in LINC01614-transduced A549 cells with indicated treatments. **C-D,** ANXA2 silencing partially reversed the effects of LINC01614 on SLC38A2 and SLC7A5 in A549 cells. The SLC38A2 and SLC7A5 levels were evaluated by RT-PCR (**C**) and western blot (**D**) (n = 3). **e**, OCR of A549 cells with indicated treatments (n = 3). **F**, ^3^H-glutamine uptake and ATP production of A549 cells with the indicated treatments (n = 3). For **A-D** and **E-F**, The mean ± s.d. are shown, and Student’s *t*-test determined *P* values. * *P* < 0.05, ** *P* < 0.01, *** *P* < 0.001. UT, cancer cells without treatment; CM, conditioned medium; Exos, exosomes; LUAD, lung adenocarcinoma.


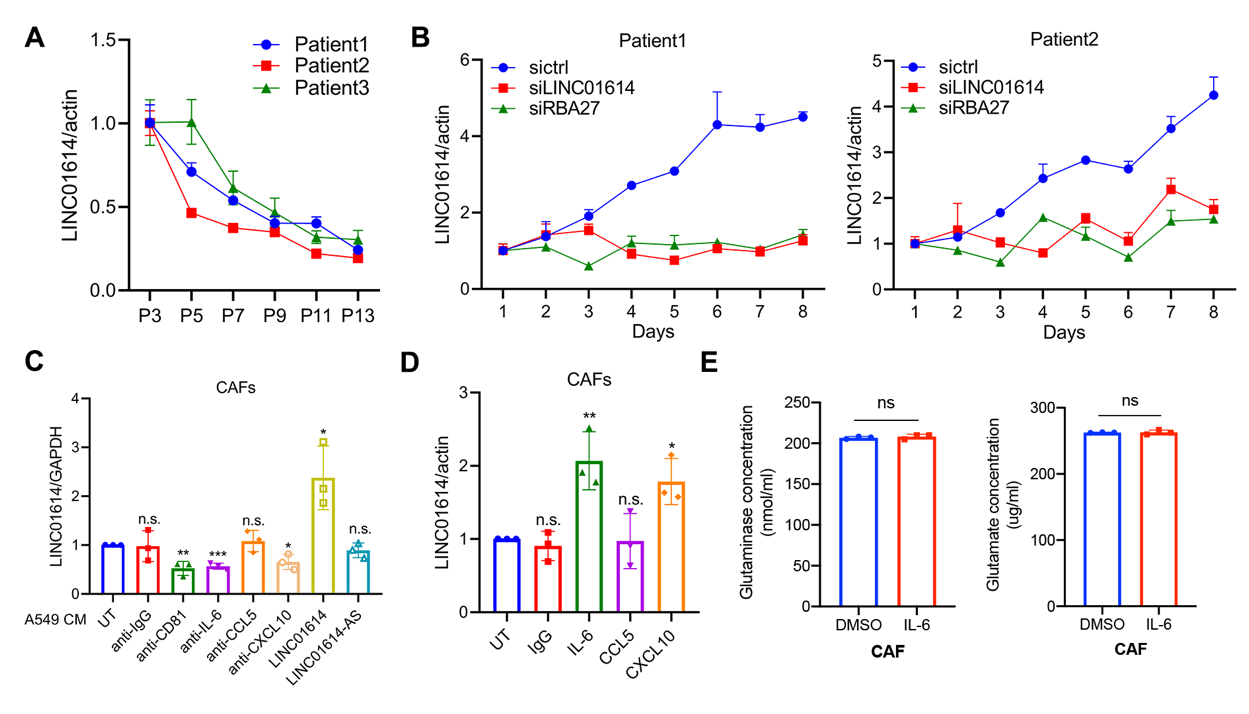


**Fig. S7 Proinflammatory cytokines secreted by LUAD cells upregulate LINC01614 in CAFs.**

**A,** LINC01614 expression in CAFs of different passages was determined by qRT-PCR. **B**, LINC01614 expression in CAFs cocultured with A549 cells for indicated times was determined by qRT-PCR (n = 3). **C,** LINC01614 expression in CAFs treated with CMs from A549 cells for indicated times was determined by qRT-PCR (n = 3). **D**, LINC01614 expression in CAFs treated with indicated recombinant proteins (n = 3). **E,** Glutaminase and glutamate content of CAFs treated with recombinant IL-6 or DMSO. For all experiments, the means ± s.d. are shown, and independent sample *t*-tests determined *P* values. * *P* < 0.05, ** *P* < 0.01, *** *P* < 0.001. UT, cancer cells without any treatment, Exos, exosomes.

**
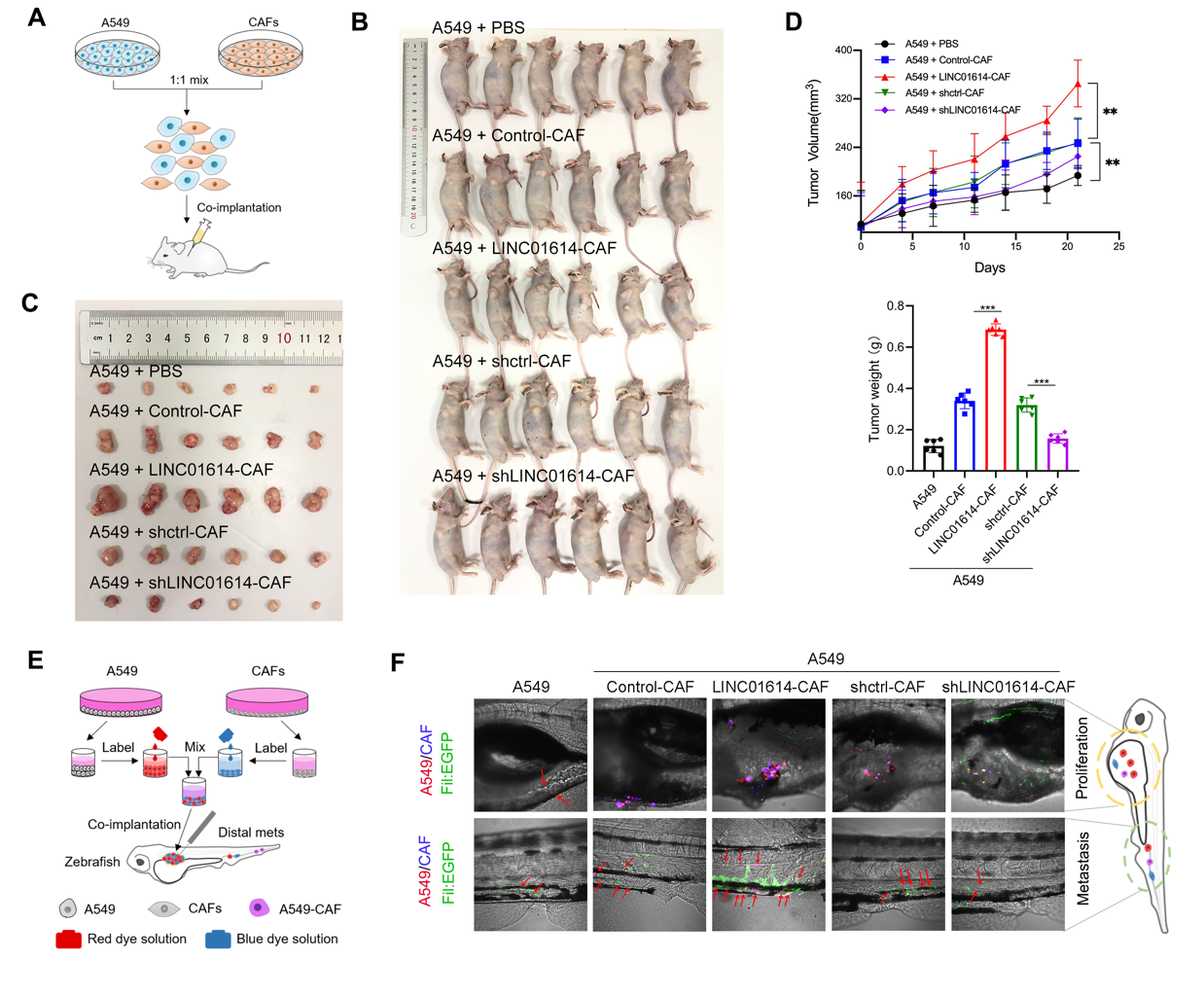
**

**Fig. S8 CAFs release LINC01614 in exosomes to enhance glutamine uptake and progression of LUAD *in* *vivo*. A-D,** A549 cells were injected alone, or co-injected with indicated CAFs at a ratio of 1:1 into a single flank of nude mice. **A,** Approach schema is illustrated. **B**, Images of tumor engraftment in nude mice. **C-D**, Tumor volume growth curves and tumor weight of nude mice. **E**, Schematic diagram of co-implantation of cancer cells and fibroblasts in zebrafish embryos (Fli1:EGFP). Cultured monolayers of A549 cells and transduced CAFs were harvested and labeled with different colors. The labeled cells were mixed and co-implanted into the perivitelline space of each zebrafish. Dissemination of A549, CAFs, and A549-CAF complexes was monitored. **F**, Representative confocal microscopy images of the dissemination of implanted cells (n = 15). Red arrowheads point to disseminated and metastatic A549 cells 72 h after implantation, yellow arrowheads point to overlapping A549 cells and CAFs. For **D**, the means ± s.d. are shown, and independent sample *t*-tests determined *P* values. * *P* < 0.05, ** *P* < 0.01, *** *P* < 0.001. UT, cancer cells without any treatment, Exos, exosomes.


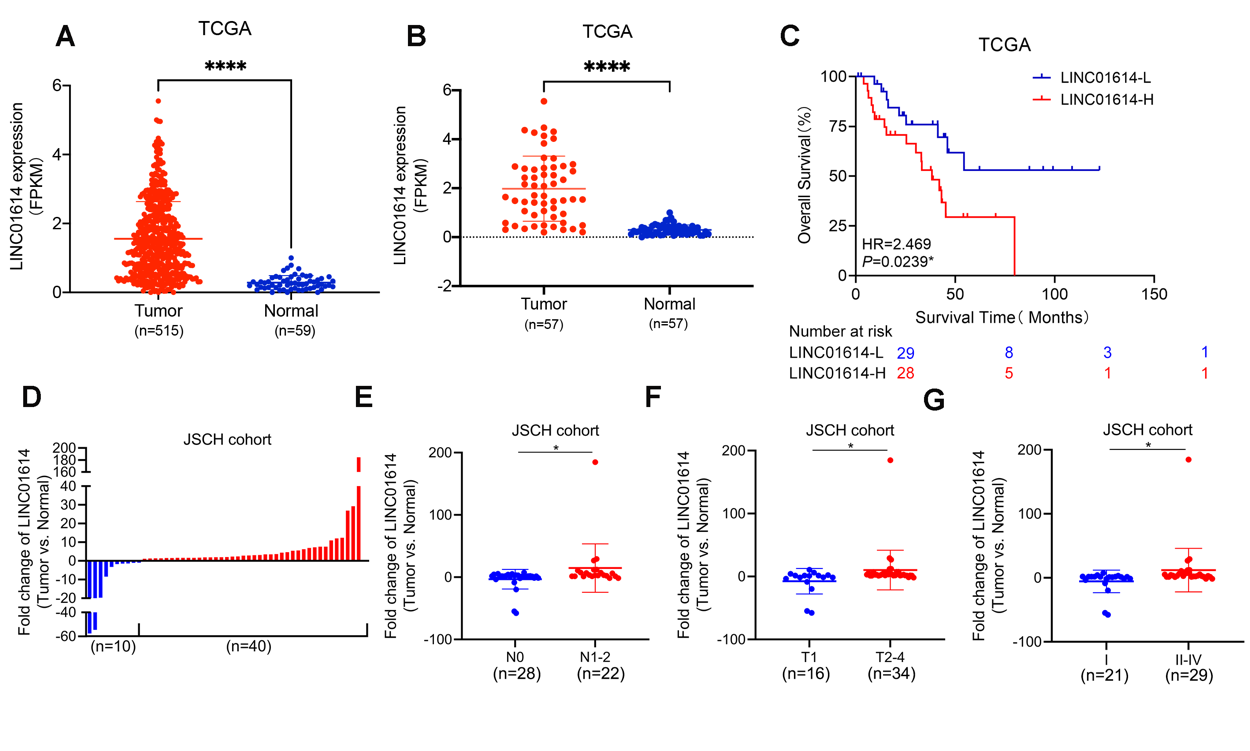


**Fig. S9 LINC01614 correlates with poor survival in patients with LUAD. A-C**, TCGA data show that LINC01614 is upregulated in LUAD tissues compared to normal lung tissues (**A-B**) and poor prognosis (**C**). **D**, qRT-PCR indicated that LINC01614 was upregulated in 40 pairs (out of 50) of LUAD tissues compared to adjacent normal tissues from the JSCH cohort. **E-G**, Expression of LINC01614 was positively associated with N stage (**E**), T stage (**F**), and overall TNM stage (**G**) in the JSCH cohort (n = 50). For **A-B** and **D-G**, the mean ± s.d. are shown, and Student’s *t*-test determined *P* values. *P* < 0.05, ** *P* < 0.01, *** *P* < 0.001. JSCH, Jiangsu Cancer Hospital; LUAD, lung adenocarcinoma.
